# A prediction framework with time-frequency localization feature for detecting the onset of seismic events

**DOI:** 10.1371/journal.pone.0250008

**Published:** 2021-04-22

**Authors:** Kanchan Aggarwal, Siddhartha Mukhopadhya, Arun K. Tangirala

**Affiliations:** 1 Department of Chemical Engineering, Indian Institute of Technology Madras, Chennai, Tamil Nadu, India; 2 Seismology Division, Bhabha Atomic Research Centre, Mumbai, Maharashtra, India; Northeastern University, UNITED STATES

## Abstract

Onset detection of P-wave in seismic signals is of vital importance to seismologists because it is not only crucial to the development of early warning systems but it also aids in estimating the seismic source parameters. All the existing P-wave onset detection methods are based on a combination of statistical signal processing and time-series modeling ideas. However, these methods do not adequately accommodate some advanced ideas that exist in fault detection literature, especially those based on predictive analytics. When combined with a time-frequency (t-f) / temporal-spectral localization method, the effectiveness of such methods is enhanced significantly. This work proposes a novel real-time automatic P-wave detector and picker in the prediction framework with a time-frequency localization feature. The proposed approach brings a diverse set of capabilities in accurately detecting the P-wave onset, especially in low signal-to-noise ratio (SNR) conditions that all the existing methods fail to attain. The core idea is to monitor the difference in squared magnitudes of one-step-ahead predictions and measurements in the time-frequency bands with a statistically determined threshold. The proposed framework essentially accommodates any suitable prediction methodology and time-frequency transformation. We demonstrate the proposed framework by deploying auto-regressive integrated moving average (ARIMA) models for predictions and the well-known maximal overlap discrete wavelet packet transform (MODWPT) for the t-f projection of measurements. The ability and efficacy of the proposed method, especially in detecting P-waves embedded in low SNR measurements, is illustrated on a synthetic data set and 200 real-time data sets spanning four different geographical regions. A comparison with three prominently used detectors, namely, STA/LTA, AIC, and DWT-AIC, shows improved detection rate for low SNR events, better accuracy of detection and picking, decreased false alarm rate, and robustness to outliers in data. Specifically, the proposed method yields a detection rate of 89% and a false alarm rate of 11.11%, which are significantly better than those of existing methods.

## Introduction

Accurate detection and P-wave picking is the most crucial step in developing early warning systems (EWS). Naturally, this topic has been of great interest to seismologists with the effect that there exists a vast amount of literature spanning a history of six decades [1–6]. Among the repertoire of existing methods, only a few have been prominently used [2, 7] owing to their simplicity and reasonable success. In principle, the requirements of any detector/picker are: (i) accuracy of detection, (ii) low false alarm rate, (iii) robustness to outliers, (iv) ability to detect in low-quality seismograms, (v) ability to handle missing data and finally (vi) ease of implementation or simplicity. None of the existing methods or any prospective method can be expected to meet all the stated requirements since they are conflicting. For instance, one may have to sacrifice simplicity for accuracy and robustness for precision, especially under low signal-to-noise ratio (SNR) conditions. Furthermore, any method is expected to take into account, to the best possible extent, additional process and measurement related aspects such as (i) weak and emergent nature of the wave, (ii) the form of P-wave varies with the source and geographical location, (iii) masking of the P-wave by natural or human-made noise and, (iv) the influence of instrument bandwidth, noise, dispersion, etc., on the recorded signal (measurement). It may be possible to devise a method that can cater to P-waves of different strengths, varying noise conditions, and geographical regions; however, the method may have to be tailored for a specific instrument. The primary motivation for this work is that existing methods are not necessarily effective under low SNR conditions, as we argue through a critical review of the prevailing literature below while also offering some useful perspectives. Moreover, there exists a rich literature on fault detection techniques, especially those that are based on predictive models, for engineering systems [8] that can be potentially tailored for enhanced P-wave onset detection. This path has been hardly explored in the seismic data analysis literature. We present, in this article, a new predictive framework with time-frequency localization capability for P-wave detection by fusing model-based fault detection techniques in process engineering and projection-based methods for signal analysis.

The proposed framework is not necessarily limited to the detection of seismic events but is rather generic in that it can be used for fault detection in other domains as well. The scope of this presentation is confined to single-channel seismic signals. A positive offshoot of the proposed framework is that it facilitates P-wave reconstruction once it has been detected. We hasten to add that this is an idea at its nascent stage and requires sufficient development and validation that is outside the scope of this work. We envisage two potential benefits of a fully developed P-wave reconstruction algorithm. Firstly, the reconstructed or the estimated (filtered) P-wave can then be used for building a library of mathematical models for P-wave. The developed empirical P -wave model can be used for detecting the onset using Kalman filters or Bayesian methods, wherein a few of the hidden variables (or the states) correspond to the dynamics of P-wave. The reconstructed P-wave can also be used in what is known as scenario matching, where the incoming P-wave can be matched with the dictionary of historical reconstructed P-waves to estimate the source parameters without using S-wave information. These are exploratory ideas that constitute topics for future studies. We return to a description of the salient features of the proposed predictive framework in the discussion to follow.

The core idea of the proposed framework is to monitor the difference of squared absolute magnitude between the data and one-step-ahead predictions in the t-f domain with the aid of a data-driven threshold. Working in a prediction framework allows the user to highlight the event even under low SNR conditions, while the t-f tool offers localization in frequency bands over desired time intervals. The localization in the t-f plane can be thought of as a zoom-in feature, which enables the user to zoom into the frequency bands of interest, thereby improving the SNR significantly since the discarded frequency bands carry away significant amounts of noise. The primary advantages of this work over the existing t-f methods are that it (i) is commensurate with the noise characteristics resulting in *minimal sensitivity to outliers* or *robust detection*, (ii) offers a more flexible frequency band selection, by decomposing both lower and higher frequencies in each level, resulting in *accurate detection*, and (iii) it allows the user to discard the noise in undesired t-f bands resulting in *improved SNR*. An important remark on the prevalent definition of SNR is in order here. However, it is a conservative metric and serves well for time-domain methods or those that work with all frequency bands. Where time-frequency localization-based methods are concerned, a band-limited SNR, defined as the ratio of the energy of t-f coefficients of the signal to that of the t-f coefficients of noise in the desired frequency band, is better suited for understanding their effectiveness. By virtue of its definition, the band-limited SNR can be high even as the standard SNR is very low. This aspect essentially explains why t-f localization methods can be superior to pure time- or frequency-domain methods.

The proposed framework is demonstrated by deploying auto-regressive integrated moving average (ARIMA) models for predictions and maximal overlap discrete wavelet packet transform (MODWPT) for the t-f decomposition of data on a toy example and 200 real-time seismic datasets. MODWPT provides two-sided decomposition making it superior to the other t-f localization techniques. A comparative analysis with STA/LTA, AIC, and WPT-AIC methods is presented to demonstrate the superiority of the proposed method. Preliminary ideas of the specific methodology being taken up were presented at a conference [9], where we demonstrated its applicability without alluding to any generic framework or the estimation of P-wave using the reconstructed signal. Moreover, [9] does not prescribe a method for t-f band selection and excludes any comparative analysis with the existing methods for different datasets with varying SNR scenarios.

The rest of this article is organized as follows. We first review the existing literature on detection and picking of P-wave onset. We then describe the proposed framework, the underlying methodology, and engage in a brief discussion on the crucial user-specified parameters that determine the effectiveness of the overall algorithm. Subsequently, we take up a case study involving a synthetic process to demonstrate the proposed framework. This is followed by an extensive application of the method to P-wave detection in 200 data sets from different stations that are characterized by a range of SNRs and earthquake magnitudes. The article concludes with a reflective summary of the work and directions for future studies.

## Literature review

Most of the existing detector / pickers are based on tracking the abrupt changes in the signal characteristics, such as amplitude, energy, frequency, higher-order statistics, etc, either in original domain or the transformed domain on the arrival of seismic event [1–4, 10–14]. Among these techniques, STA / LTA (short-term average / long-term average) [2] and its variants [15, 16] are widely used for the ease of computation and online implementation. The working principle of STA/LTA methods is based on comparing the ratio of energies in a short and long window with a threshold. These methods result in a high false alarm rate because of the way they differentiate noise from the signal. This is an inherent drawback to all the time-domain techniques due to ignorance of P-wave frequency content. Moreover, since only time properties are used, these methods are sensitive to outliers. The inherent limitations of time-domain methods inspired the development of a handful of frequency-domain methods, where frequency or energy is used as a feature to detect the P-wave onset [1, 3, 12]. However, these methods did not receive wide attention in practice because they use the entire frequency range (0 to Nyquist frequency), thus not providing effectively any specific advantage over their time-domain counterparts. The true benefit is realized by only combining the merits of working in both domains. Thus, researchers developed time-frequency detectors taking into account the time properties and frequency information of the seismic signal. Features in mixed-domain, especially using time-frequency (t-f) tools such as continuous and discrete wavelet transform (CWT, DWT) [13, 14], short-time Fourier transform (STFT) [5] and empirical mode decomposition (EMD) [6], have been widely used in this regard. The generic idea underlying these methods involves projecting the data into a t-f (or time-scale) domain and search for the feature either in the mixed-domain or in the reconstructed signal. Recent advancements in these methods combine the benefits of time-domain methods with the t-f tools [7, 17, 18] to improve the detection rate for low SNR seismic signals. Irrespective of the way in which the t-f tools have been used in the seismic literature, their use has only involved decomposing the lower frequency content (approximations) at each scale, thereby limiting the localization to lower frequency ranges. This one-sided decomposition may not always contain the P-wave information.

Early 1990s witnessed the development of pattern-based methods to detect the weak events embedded in noise [19–22]. These methods compare the similarity between the earthquake signal and the event pattern (template). [23] proposed a method to detect local events embedded in locally stationary background noise. Most of the pattern-based methods either use a fixed template of P-wave or assume that the signal under consideration exhibit specific features. Moreover, various other factors, such as complicated source mechanisms and the dependency of P-wave frequency on source-station location, limit the applicability of these detectors to exceptional situations such as aftershocks and repeating sources.

In the recent years, detectors based on machine learning / deep learning (ML/DL) have been proposed [24, 25]. The method of [24] delivers probabilities associated with the existence of an earthquake event and two different seismic phases for each time point by using encoders that intrinsically capture the temporal dependencies in seismic data. The decoders consist of carefully designed set of deep learning network models comprising tens of layers and about 372000 tunable parameters for detecting and picking the seismic phase arrivals. The method proposed by [25] utilizes capsule neural network (CapsNet) to pick the P-wave arrival. The CapsNet consists of three main layers for classifying the data as noise and earthquake signal, followed by the extraction of P-wave arrival time. In both the works, the trained complex models are shown to result in highly accurate detection and picking performance; however, the approaches are not only data extensive (in the sense that huge amounts of data are required to train the models) but also involves the design of a highly complex model architecture. Finally, despite their high levels of complexity, the construct of these models may result in an unacceptable level of false alarm rates. A remedy suggested by [24] is to explicitly incorporate the spectral features of seismic signals.

It is evident that the existing P-wave onset detection literature can be classified in a few different ways depending on the viewpoints taken. A few useful perspectives are obtained by choosing to classify the literature based on the approach adopted, namely, (i) feature-based, (ii) pattern recognition-based, and (iii) model-based methods. The bulk of existing detection methods are feature-based, while the pattern-based and model-based approaches are seen only in a handful of methods. Regardless of the approach taken, the implementation can be directly on the raw data (time-domain), or in the transform domain (usually frequency domain), or in the mixed (e.g., time-frequency) domain. An advantage of working in the mixed-domain is that the signal characteristics can be captured simultaneously in a time interval and frequency band. Consequently, because the seismic signal is a multi-scale (each scale being associated with a frequency band) signal, P-wave is better highlighted in the mixed-domain compared to the time-domain signal, especially for low SNR conditions. Moreover, since signal features are concentrated in a relatively smaller region (lower frequency ranges), the undesired noise in the higher frequency ranges can be ignored, resulting in improved detection. It is apparent, therefore, that time-domain implementations are the most widely used, followed by relatively more recent works in the mixed-domain [13, 17] while transform-domain implementations have received very little attention. Within the class of mixed-domain implementations, there exist a subset of methods that work with *projections* (on to a chosen set of basis functions or atoms such as wavelets) or *coefficients*, while the remaining subset uses *reconstructed* or *filtered* signals, where the particular transform is used essentially as a filter to select known components of the seismic measurements. A significant advantage of projection-based approaches over the reconstruction-based approaches is that the desired features in the signal are highly localized in the projection-domain as compared to the time-domain (reconstructed signal). Moreover, projection coefficients are not as strongly correlated as the reconstructed signal resulting in the comparatively sparse representation in the projection-domain.

Model-based methods offer the privilege of taking a predictive approach to the detection problem, which is not present in model-free methods. These approaches have been extensively used in process engineering, especially for fault detection [8], ideas from which, however, remain to be fully exploited in seismic data analysis. A handful of methods, widely known as AR-AIC pickers, appeared in the late 1980s and 1990s built time-series models and tracked the associated Akaike-Information criterion (AIC), [26–28]. AR-AIC pickers outperform the STA/LTA detectors; however, these methods assume that noise and event are locally stationary and do not provide proper justification for using AR models. Furthermore, it may not be technically appropriate to model P-wave as a stochastic process. In the aforementioned works, the general tendency is to examine the AIC and not the predictions, thereby not truly leveraging the benefits of a model-based approach. A significant benefit of working with predictive approaches is that the measurements are decomposed into a predictable component and a prediction error (what is unexplained by the model). The first consequence of this decomposition is that the prediction error has a lower variance than that of the measurement—the extent to which it is lower depends on the predictable component. The higher the strength of the predictable component in the measurement, the lower is the variance of prediction error. Secondly, any feature that is not contained in the historical record will appear in the prediction error along with the unpredictable portion of the measurement. It is relatively easier to detect the “new” signals in the prediction errors than in the original measurement because the signal-to-noise ratio is enhanced significantly in the prediction error domain. Thus, a predictive approach is naturally better positioned in handling low SNR measurements than the methods that are not in the predictive framework. Needless to say, the goodness of a prediction-based method depends on the model quality. To this end, the authors of this work, in a separate study, have developed a systematic methodology for building statistically appropriate time-series models for seismic noise [29]. This work essentially builds on the models developed and exploits the advantages of a model-predictive framework, especially for handling low SNR situations.

## Proposed framework

**Fig 1:**
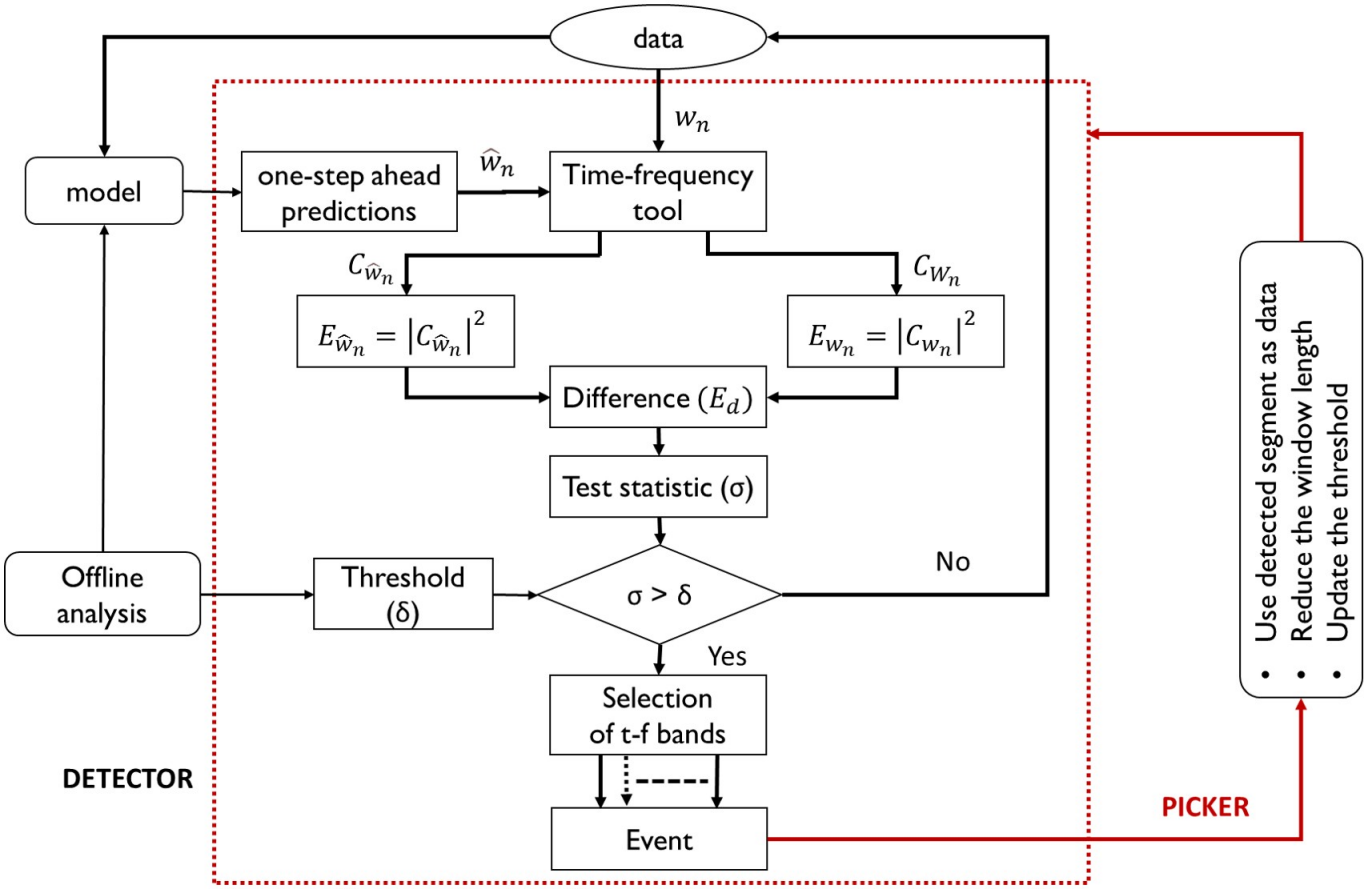
Proposed framework for P-wave detection and picking that accommodates any modeling technique (for prediction) and a t-f tool (for decomposition).

This section presents the details of the proposed predictive framework for accurate detection and picking of P-wave in the seismic signal using the vertical channel data. The proposed framework essentially consists of two parts, as depicted in Fig 1, one that results in accurate detection, and the other responsible for the accurate picking of P-wave. The predictive models are developed in time-domain while the detection is carried out in the t-f domain with the aid of a t-f transformation tool. The principle underlying detection using the proposed method is as follows. For a given time window, the estimated noise model would result in optimal predictions if the window contains the background noise. On the contrary, predictions will be poor if the window contains the event data. Further, as a consequence of working with predictions in the t-f domain, an abrupt change in the difference of squared absolute magnitude of t-f coefficients occurs on the arrival of the seismic event. This change is highlighted with the aid of a threshold to alert the detection of an event. Post detection, picking (identifying the time of event onset) is carried out by a ranking of the highlighted t-f bands noting that the difference of squared absolute magnitude is more prominent in certain t-f bands pertaining to the P-wave frequency than in other bands. This ranking not only facilitates picking but also the reconstruction of the P-wave signature, which as aforementioned, is novel and potentially useful in advanced stages of seismic data analysis. The proposed framework is graphically depicted in Fig 1. This schematic describes the steps implemented on a window of data.

Assume initially that *N* observations (corresponding to a duration of *T* = *NT*_*s*_ sec, where *T*_*s*_ is the sampling interval) of the seismic signal *y*[*k*] are available online, where *N* is a user-defined parameter (see below for details). The procedure consists of the following steps:

1. Construct a window of data
  Standing at the instant *k* = *N* + (*n* − 1)*S*, where *S* is a user-defined sliding parameter and *n* is the window index (initially set to 1), construct a window of *N* past observations as follows:
  $$\begin{array}{c} w_{n}\left[k\right]≜{\left[\begin{array}{cccc} y[k] & y[k-1] & \ldots & y[k-(N-1)] \end{array}\right]}^{T} \end{array}$$ (1)
  whereby **w**_*n*_ is a vector of size *N* × 1.
2. Compute one-step ahead predictions
  Using the time-series model developed offline, compute one-step ahead predictions of observations in the window,
  $$\begin{array}{c} {\hat{w}}_{n}\left[k|k-1\right]={\left[\begin{array}{ccc} \hat{y}\left[k|k-1\right] & \hat{y}\left[k-1|k-2\right] & \cdots \hat{y}\left[k-\left(N-1\right)|k-\left(N-2\right)\right] \end{array}\right]}^{T} \end{array}$$
3. Project data and predictions using the t-f tool
  Decompose both the incoming data, **w**_*n*_[*k*] and the predictions, ${\hat{w}}_{n}$[*k*∣*k* − 1] in t-f domain up to a desired level *L* using a suitable t-f tool. At each level *l* (*l* = 1, 2, ⋯, *L*), t-f coefficients are denoted by **C**(*l*, *ω*_*c*,*i*_): {*C*(*l*, *ω*_*c*,*i*_, *k*)}, where at level *l*, **C**(*l*, *ω*_*c*,*i*_) is 2^*l*^ × *N* matrix, *ω*_*c*,*i*_ represents the center frequency of the *i*^th^ (*i* = 1, ⋯, *B*_*l*_) frequency band and *k* represents the time instant. Note that the maximum index of frequency band at each level *l* is *B*_*l*_ = 2^*l*^.
4. Compute the squared absolute magnitude in t-f bands
  The squared absolute magnitude of projections of data and predictions in the *i*^th^ t-f band at each level *l* is defined as:
  $$\begin{array}{ccc} E_{w_{n}}\left(l,\omega_{c,i}\right) & = & |C_{w_{n}}\left(l,\omega_{c,i}\right){|}^{2} \end{array}$$ (2)
  $$\begin{array}{ccc} E_{{\hat{w}}_{n}}\left(l,\omega_{c,i}\right) & = & |C_{{\hat{w}}_{n}}\left(l,\omega_{c,i}\right){|}^{2} \end{array}$$ (3)
  where $C_{w_{n}}$ and $C_{{\hat{w}}_{n}}$ are the projections of signal and predictions respectively and *ω*_*c*,*i*_ is the center frequency as defined earlier.
5. Compute mean absolute deviation (μAD)
  In the *n*^th^ window **w**_*n*_[*k*], the mean absolute deviation of difference in squared absolute magnitudes between the projections of data and predictions in the *i*^th^ t-f band at each level *l* is defined as:
  $$\begin{array}{c} {\mu AD}_{l,i}\left(n\right)=\text{mean}|E_{d}\left(l,\omega_{c,i}\right)-\text{median}\left(E_{d}\left(l,\omega_{c,i}\right)\right)| \end{array}$$ (4)
  where, the difference, **E**_*d*_, is given by
  $$\begin{array}{c} E_{d}\left(l,\omega_{c,i}\right)=E_{w_{n}}\left(l,\omega_{c,i}\right)-E_{{\hat{w}}_{n}}\left(l,\omega_{c,i}\right) \end{array}$$ (5)
  *μAD* is known to be a biased estimate of standard deviation, therefore, we work with a bias-corrected *μAD*,
  $$\begin{array}{c} {\mu AD}_{l,i}\left(n\right)=K\times \text{mean}|E_{d}\left(l,\omega_{c,i}\right)-\text{median}\left(E_{d}\left(l,\omega_{c,i}\right)\right)| \end{array}$$ (6)
  where, *K* = 1.25 is the correction factor that is numerically determined for the random variable **E**_*d*_ through Monte-Carlo simulations (see S1 Appendix in S1 File). Note that *E*_*d*_(*l*, *ω*_*c*,*i*_) approximately follows a Gamma distribution, which is determined through an empirical fit.
6. *Compare the μAD in selected t-f bands with the respective thresholds* to detect the abrupt changes.
  Let ***δ***_*l*,*i*_ be the vector of *B*_*l*_ thresholds for background noise in the respective t-f bands. If the window, **w**_*n*_[*k*] contains only the seismic noise then ***μAD***_*l*,*i*_(*n*) in the t-f bands will not vary as *k* varies, however, ***μAD***_*l*,*i*_(*n*) increases abruptly in certain t-f bands on the arrival of P-wave in **w**_*n*_[*k*].
7. Make a decision
  Flag those bands for which ***μAD***_*l*,*i*_(*n*)>***δ***_*l*,*i*_ across all bands and levels.
  - *No Detection*: In the case where no event is detected, set the detection flag to 0, slide the window with sliding length (*S*) and proceed to step 8. Selection of *S* is based on various factors such as SNR, the sampling rate of data and type of seismic event, etc.
  - *Detection*: If an event is detected, implement the following
    - Set the detection flag to 1.
    - Pick the onset of the event precisely by reducing the window length, and repeating the steps for the detected segment of data. The new working window length in the detected segment also depends on SNR, sampling rate, etc of the data. Typically, a window of length 1-2 seconds is used for picking the event in the detected segment because the P-wave is a short duration signal.
    - Select the “best” t-f bands. The bands that are highlighted in Step 6 (on the arrival of P-wave) are ranked in the ascending order of the detection onset time and are selected for *picking* the onset of P-wave. Selecting the packets (bands) of interest dynamically as against working with a pre-determined set of bands from historical data analysis makes the approach adaptive to the form of P-wave, the source-station location, etc. In addition, since the selected t-f bands generally span the P-wave frequency, they can be used for reconstructing the time-domain signature of the P-wave, which in turn, can be used for at least two different advanced seismic data analysis (refer to Introduction section for a brief outline). This, we believe, is a positive outcome of the proposed methodology.
8. *Repeat the above steps* for online detection of the seismic event.

The performance of the proposed method depends on various parameters or variables. A summary with a brief description and optimal values (for user-defined parameters) of these parameters is presented in Table 1. The optimal values of data-driven parameters vary with datasets and therefore are represented by DD (data dependent) in the table. The influence of these parameters on the performance of the proposed algorithm is discussed later in the following sections.

**Table 1:** List of parameters used by the algorithm with brief description and values used in the study.

Parameter	Nature	Description	Value
*N*	user-defined	Length of working window (scalar)	240
*S*	user-defined	Sliding length (scalar)	5
***δ***	data-driven	Threshold (vector)	DD
*L*	user-defined	Level of decomposition (scalar)	4 for 20 sps data
			5 for 40 sps data
t-f bands	data-driven	Selected t-f bands	DD

The efficacy of the proposed framework is demonstrated using a widely used time-series model known as the ARIMA model for prediction purposes, and the a well-established t-f tool, namely, the wavelet-transform based MODWPT as the t-f tool for decomposing the data in t-f bands. ARIMA models are linear time-series models that essentially captures the integrating effects by developing ARMA models on the differenced series [30]. The governing equation of an ARIMA(*p*,*d*,*m*) model is given by:

$$\begin{array}{c} (1-\sum_{i=1}^{p}\phi_{i}q^{-i}){\left(1-q^{-1}\right)}^{d}y\left[k\right]=(1+\sum_{j=1}^{m}\theta_{j}q^{-j})e\left[k\right] \end{array}$$ (7)

where, *q*^−1^ is the backshift operator, *ϕ*_*i*_ and *θ*_*j*_ are the AR and MA coefficients of order *p* and *m* respectively, *d* is the degree of differencing and *e*[*k*] is a zero-mean Gaussian white-noise (temporally uncorrelated) of variance $\sigma_{e}^{2})$. Estimation of ARIMA models involves identification of the model orders *p*, *d*, *q*, where *d* is the degree of differencing, and *p* and *q* represents the order of AR and MA coefficients, *p* + *q* model parameters and variance of the driving force. The unknowns are estimated using a maximum likelihood estimation algorithm.

**Fig 2:**
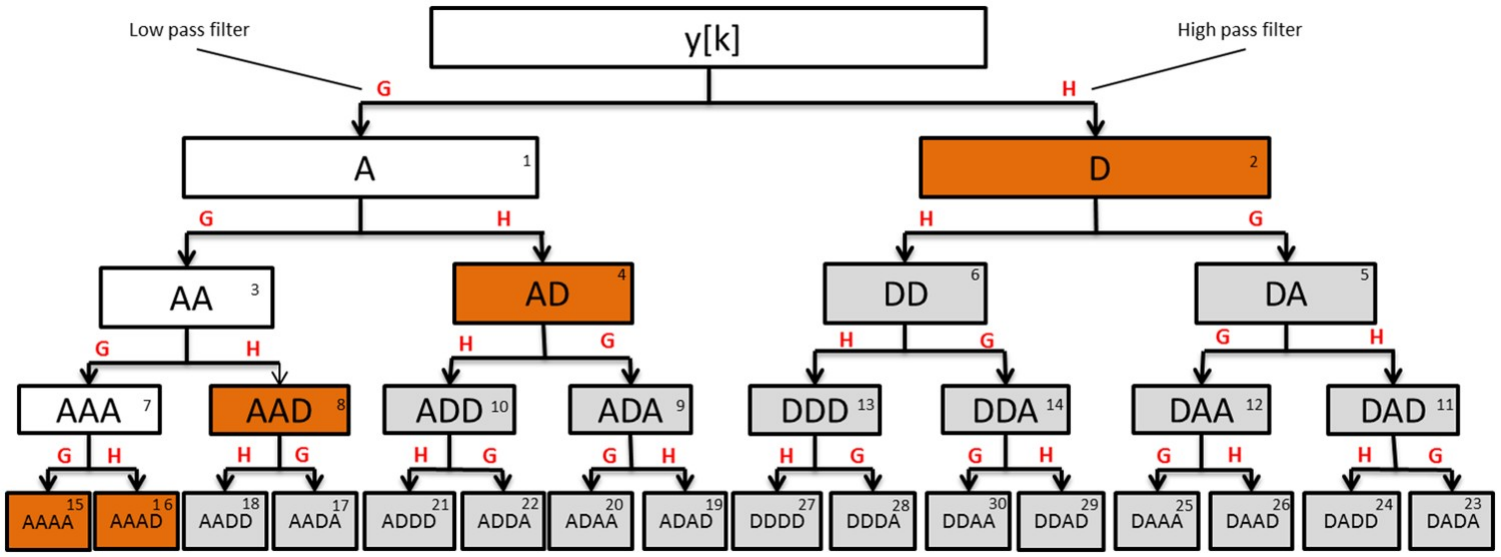
Schematic of MODWPT decomposition upto level 4. ‘A’ and ‘D’ refers to approximation and detail respectively. Integer in the top-right corner of each box indicates the packet number in increasing frequency order.

MODWPT is the generalization of wavelet transform in which signal is decomposed into low and high-frequency bands, and both the low-frequency (approximations) and high-frequency (details) coefficients are further decomposed into sub-frequency bands at each level [31]. Fig 2 shows the MODWPT schematic, where each box is referred to as a wavelet packet (also called as a node). Each packet corresponds to a particular frequency band. Frequency mapping for MODWPT packets up to level 4 is given in Table 2 for a sampling frequency of 20 Hz. The range of frequencies spanned by each band for a different sampling frequency *F*_*s*_ can be re-calculated in a straightforward manner as 0 − *F*_*s*_/4, *F*_*s*_/4 − *F*_*s*_/2 at Level 1 and so on.

**Table 2:** Frequency mapping for MODWPT packets. Level indicates the level of decomposition and the frequencies are reported for a signal sampled at 20 Hz.

Level	Node	Frequency (Hz)	Level	Node	Frequency (Hz)
Level 1	1	0-5	Level 4	16	0.625-1.25
	2	5-10		17	1.25-1.87
Level 2	3	0-2.5		18	1.87-2.5
	4	2.5-5		19	2.5-3.126
	5	5-7.5		20	3.126-3.75
	6	7.5-10		21	3.75-4.376
Level 3	7	0-1.25		22	4.376-5
	8	1.25-2.5		23	5-5.625
	9	2.5-3.75		24	5.625-6.25
	10	3.75-5		25	6.25-6.876
	11	5-6.25		26	6.876-7.5
	12	6.25-7.5		27	7.5-8.126
	13	7.5-8.75		28	8.126-8.75
	14	8.75-10		29	8.75-9.376
Level 4	15	0-0.625		30	9.376-10

A remark on the data requirements and model complexity for the chosen predictive model class is in order here. About 10 minutes of data is sufficient to obtain a reasonably good estimate of the model. Secondly, the number of tunable parameters is restricted to about 20. Thus, in terms of both, data requirements and model complexity, the chosen model is several orders lower as compared to the DL-based methods. This presents a feasible opportunity to update the model as one obtains streaming data. Thirdly, the data does not have to be subjected to labelling and other pre-processing steps as the prevailing DL models necessarily require.

## Results and discussions

In this section, the performance of the proposed method is illustrated with the help of synthetic data with varying SNR and 200 real-time seismic datasets characterized by a range of SNRs and earthquake magnitude.

### Application to synthetic datasets

The primary purpose of this section is to illustrate the application of the proposed framework to detect a short-lived signal for varying SNR with the help of synthetic data.

#### Data generation

Synthetic data *y*[*k*]: {*k* = 1, 2, …, 50000} can be represented as

$$\begin{array}{c} \underset{\text{measurement}}{y[k]}=\underset{\text{signal}}{s[k]}+\underset{\text{colored}\;\text{noise}}{v[k]} \end{array}$$ (8)

where, *s*[*k*] represents the desired short-lived signal generated using an exponentially decaying sine wave, and *v*[*k*] represents the colored noise. The signal *s*[*k*] is given by

$$\begin{array}{c} s\left[k\right]=\{\begin{array}{c} ae^{-bk}\sin\left(2\pi f_{0}k\right),\;20000<k\leq 20040\\ 0,\;\text{otherwise} \end{array} \end{array}$$ (9)

where, *f*_0_ is fixed at 1.8 Hz, signal *s*[*k*] is sampled at 20 samples per second (sps) and the values of *a* & *b* are varied to change the SNR. Details of different values of *a* and *b* along with the SNR and band-limited SNR are summarized in Table 3. The noise sequence *v*[*k*] is generated using a realistic time-series model developed for real-time seismic noise from TSUM station, Namibia, Africa. Data generating process for *v*[*k*] is given by

$$\begin{array}{ccc} v[k] & = & \frac{1}{1-q^{-1}}v^{{}^{\prime }}\left[k\right] \end{array}$$ (10)

$$\begin{array}{ccc} v^{{}^{\prime }}\left[k\right] & = & \frac{1-0.39q^{-1}-1.7q^{-2}+1.03q^{-3}+0.82q^{-4}-0.75q^{-5}}{1-0.43q^{-1}-1.3q^{-2}+0.02q^{-3}}\ldots \\ & & \ldots \frac{+0.03q^{-6}+0.09q^{-7}-0.105q^{-8}}{+0.73q^{-4}}e\left[k\right] \end{array}$$ (11)

where *e*[*k*] is a zero mean Gaussian white noise with variance *σ*^2^ = 155.78.

**Table 3:** Details of parameters used to generate synthetic data.

	Case 1	Case 2	Case 3	Case 4	Case 5
**a**	100	110	180	200	500
**b**	0.8	0.8	0.8	0.8	0.8
**f_0_ (Hz)**	1.8	1.8	1.8	1.8	1.8
**SNR**	0.021	0.049	0.46	1.8	2.09
**Band-limited SNR**	0.209	0.29	1.46	10.76	26.55

#### Results for synthetic data

**Fig 3:**
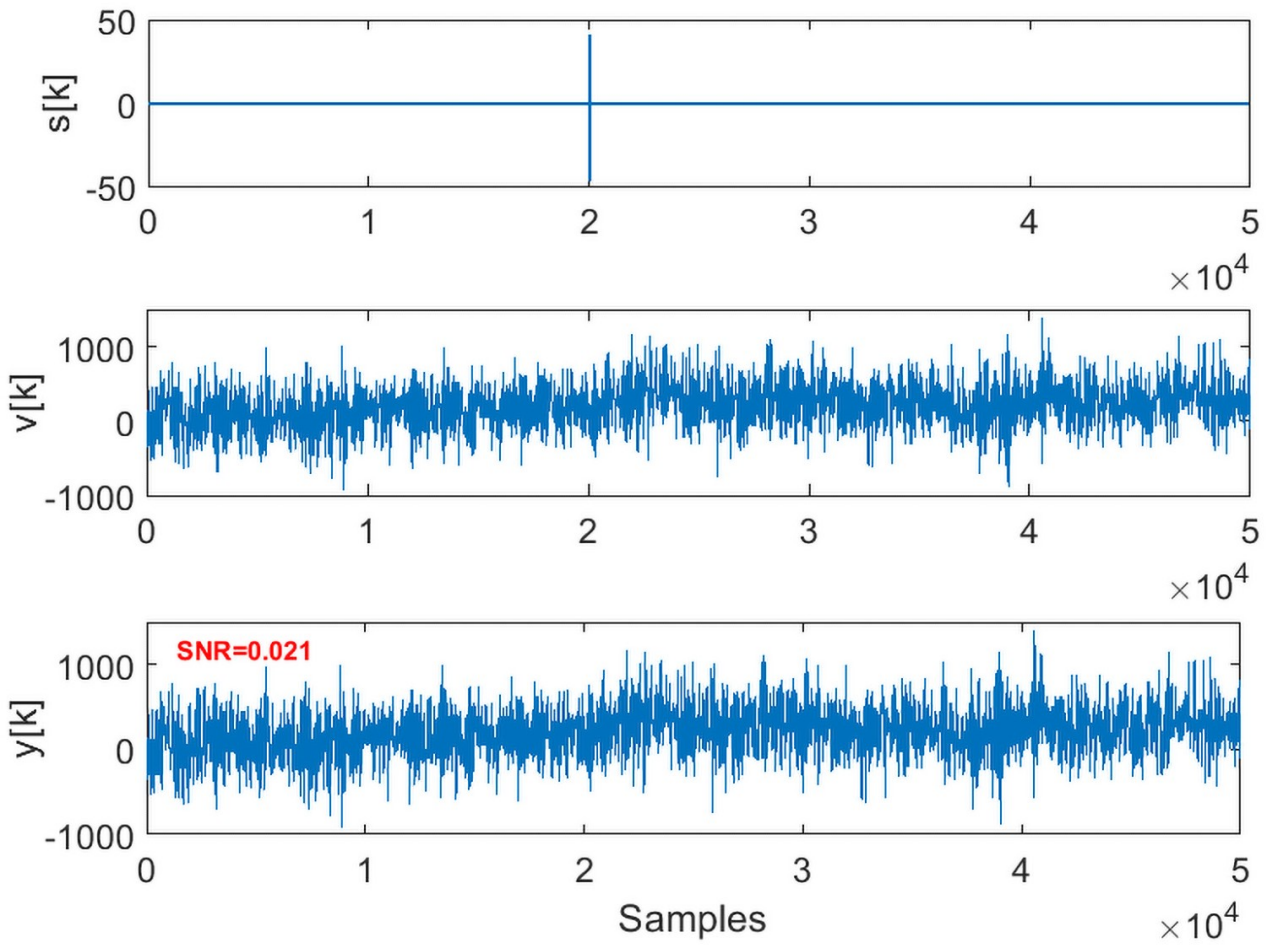
Low SNR synthetic data with a short-lived event embedded in noise.

**Fig 4:**
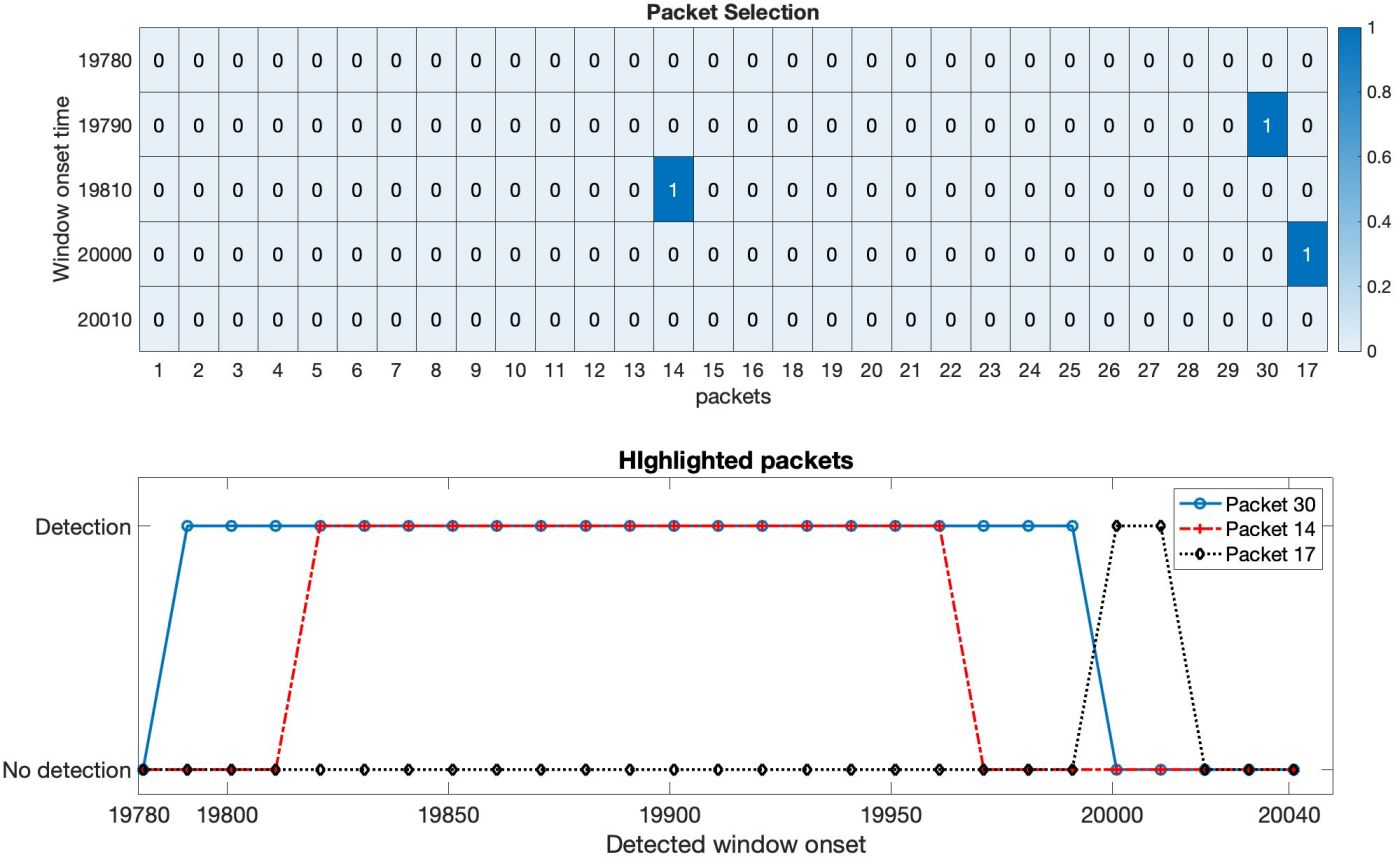
Packet selection. The plot on top depicts the selected packets on the fly based on the ranking of highlighted packets. Y-axis represents the window onset time in the ranked order (top to bottom). The presence of 1 indicates detection, and 0 represents no detection. The duration of highlighted packets is shown in the bottom plot, where the X-axis represents the onset of the working window.

**Fig 5:**
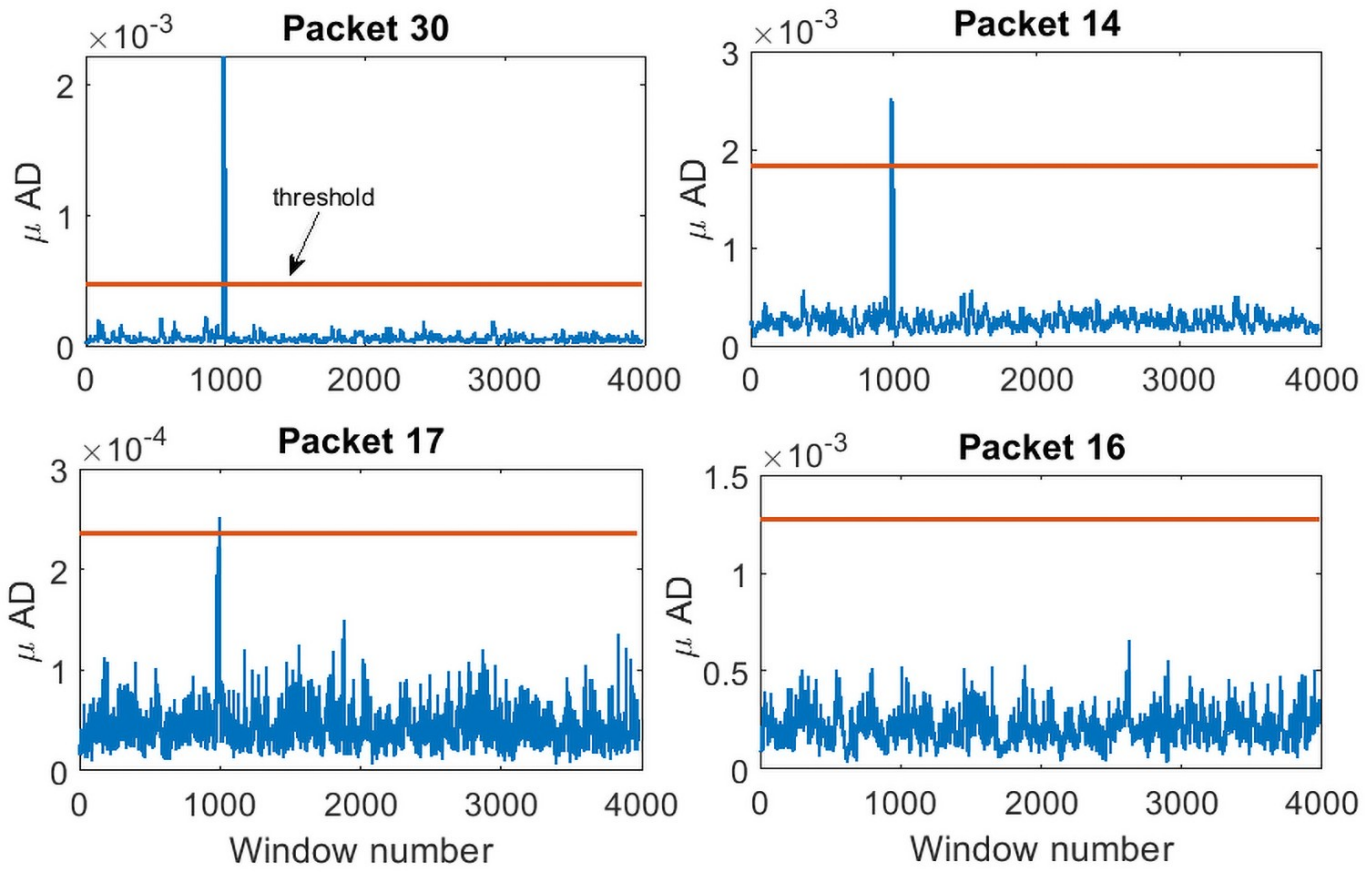
*μAD* in the highlighted packets. There is a sudden increase in the *μAD* in certain packets (30, 14 and 17) while in other packets (16 is shown in the figure, but it holds for the rest of the packets), *μAD* is always below the threshold.

Given the measurements *y*[*k*], the method aims to accurately detect and pick the onset of signal *s*[*k*]. Implementation of the proposed framework is illustrated in detail for the case 1 data, with the results being summarized for the rest of the cases. Case 1 data (SNR = 0.021) is shown in Fig 3. Initial 10000 data points are used to estimate the predictive ARIMA(4, 1, 8) model and calculate the threshold in each packet. Using a sliding window of 240 samples with sliding of 10 samples, both **w**_*n*_[*k*] and ${\hat{w}}_{n}\left[k\right]$ are decomposed in the time-frequency domain using Daubechies 4 (dB4) wavelets via MODWPT. The *μAD* of the difference of squared absolute magnitude of MODWPT coefficients of **w**_*n*_[*k*] and ${\hat{w}}_{n}\left[k\right]$ in all the packets is compared to the threshold in the respective packets. On the arrival of signal *s*[*k*], certain t-f bands (packets 8, 18, 17 and 30 for case 1 data) are highlighted. Fig 4 shows the selected packets in the ascending order of the detection onset. Packets that detect the onset of *s*[*k*] with minimum samples of *s*[*k*] in **w**_*n*_[*k*] are given higher priority. It can also be observed that when the moving window does not contain any event, no packets are highlighted (represented by 0 value in the top plot of Fig 4). The presence of 1 indicates the highlighted packets. The *μAD* in different packets along with the threshold is shown in Fig 5. For picking purposes, a sliding window of 20 samples with a sliding of 1 sample is used in the detected segment for the selected packets. For the case 1 data, the method picks the onset of *s*[*k*] with an error of 3 samples.

**Fig 6:**
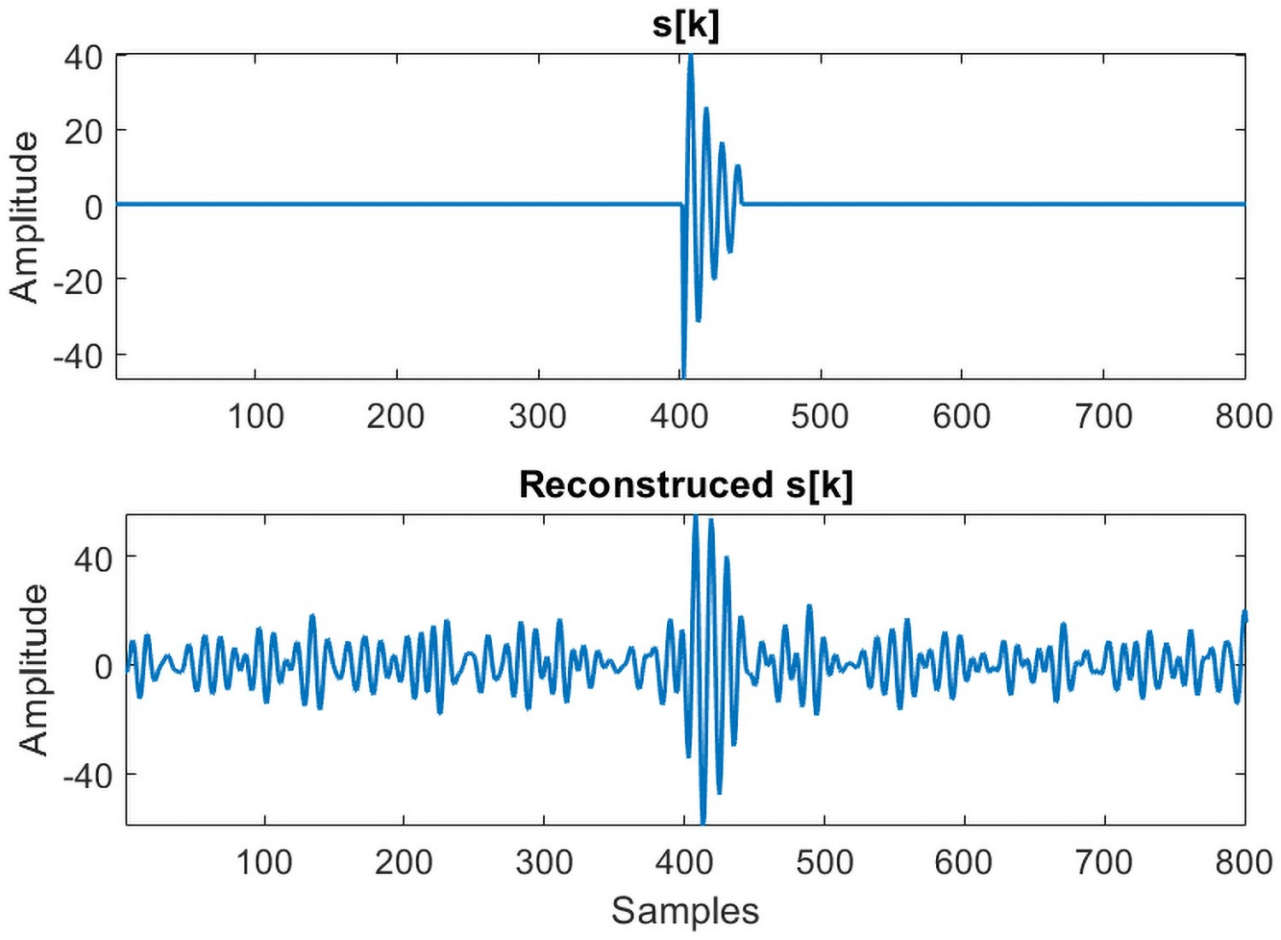
Reconstructed signal. Amplitude of the reconstructed signal is smaller than the original *s*[*k*].

Fig 6 shows the reconstructed signal using the selected packets. For reconstruction purpose, data from *k* = 19500 to *k* = 20500 is used. The onset of signal *s*[*k*] is clearly visible in the reconstructed signal even for the low SNR case. The main purpose of reconstructing the signal is to obtain a mathematical model for signal *s*[*k*] from the measurements. This can be extremely useful in obtaining a P-wave model, which is otherwise missing in the literature.

**Fig 7:**
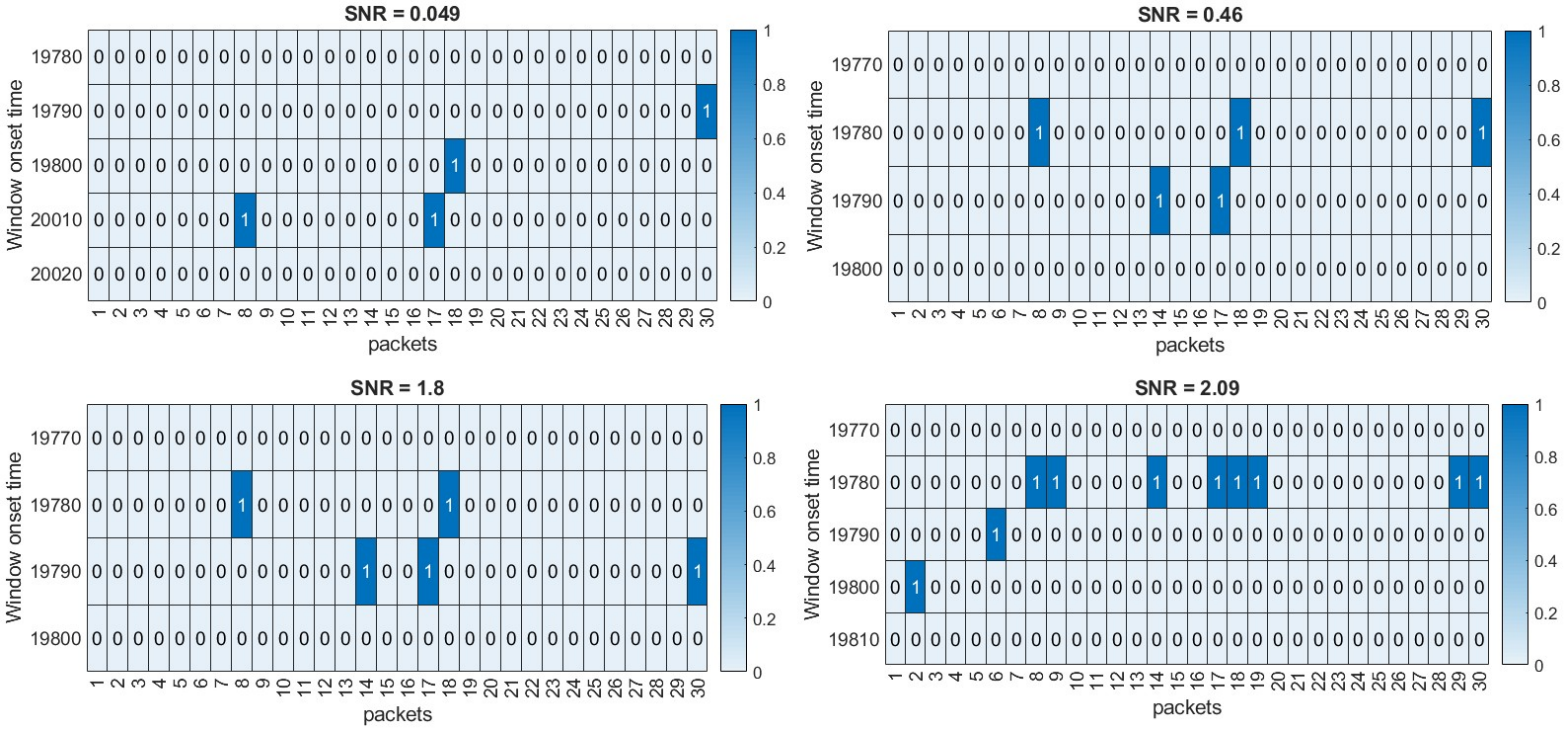
Selected packets for different SNR cases. Number of highlighted packets increases as the SNR increases.

Similarly, we have applied the proposed method to the rest of the four cases, and it is observed that the method detects the onset of *s*[*k*] even for extremely low SNR cases. The reason for accurate detection, especially for low SNR scenarios, is that the signal is highly localized in the t-f domain. Further, the band-limited SNR is higher than the standard SNR for all the cases, reported in Table 3. The latter looks at a frequency band spanning from 0 to Nyquist frequency (10 Hz for this case) while the former spans the frequencies in the desired band (from 1.25 to 2.5 Hz). This explains the improved performance of the proposed method even for extremely low SNR cases. It is also observed from Fig 7 that with the increasing SNR, more packets are highlighted.

### Application to real-time seismic data

The ability and efficacy of the proposed framework to detect the onset of P-wave is illustrated using 200 real-time seismic datasets of varying magnitudes and signal-to-noise ratio (SNR). Data is collected from four different stations, ANMO (New Mexico, US), ANTO (Turkey), MAJO (Matsushiro, Japan), and TSUM (Namibia, Africa), and is freely available on Incorporated Research Institutions for Seismology (IRIS). A link to the website from where the data sets can be directly downloaded is being provided in S2 Data in S1 File. Out of 200, 173 datasets contain seismic events while 27 are event-free, i.e., they do not contain any seismic event (noise). Magnitude of datasets is classified into four categories, (i) Mag ≤2.5, (ii) 2.5< Mag ≤4, (iii) 4< Mag ≤6, and (iv) Mag >6 to evaluate the performance of the proposed method. Details of all the datasets are summarized in Table 4 where SNR_BL_ stands for band-limited SNR. For real-time datasets, SNR_BL_ is the ratio of the energy of t-f coefficients of the signal in the detected segment to that of the t-f coefficients of noise in the highlighted t-f bands. We have also compared the performance of the proposed framework with STA/LTA, AIC picker, and DWT-AIC picker. Time reported on the IRIS website is considered as the reference time.

**Table 4:** Seismic data. Details of different datasets downloaded from IRIS.

Station	Magnitude	Counts	SNR (dB)	SNR_BL(*dB*)_	Station	Magnitude	Counts	SNR (dB)	SNR_BL(*dB*)_
ANMO	M ≤ 2.5	9	-.38 to.37	.09 to 16.03	ANTO	M ≤ 2.5	10	-.1 to.09	.08 to 3.2
	2.5 <M ≤ 4	14	-.8 to.82	.03 to 17.35		2.5 <M ≤ 4	14	-1.4 to 3.9	.02 to 2.4 × 10^4^
	4 <M ≤ 6	10	-2.6 to 17.2	.13 to 104.8		4 <M ≤ 6	10	.18 to 38.5	5.1 to 9.6 × 10^3^
	M > 6	11	9 to 61	10 to 2.5 × 10^6^		M > 6	10	-2.15 to 69.6	2.1 to 7.7 × 10^6^
	no event	27	-	-	TSUM	M ≤ 2.5	15	-1.2 to.08	.09 to 15.9
MAJO	2.5 <M ≤ 4	19	-.7 to 1.2	.35 to 374.8		2.5 <M ≤ 4	14	-1.4 to.02	.1 to 8.6
	4 <M ≤ 6	10	-.22 to 40	.14 to 1.38 × 10^4^		4 <M ≤ 6	11	-11.6 to 27.3	.47 to 1.07× 10^3^
	M > 6	11	10.3 to 71.7	35.8 to 1.17 × 10^6^		M > 6	3	32 to 37.5	(.64 to 4.61) × 10^4^

**Fig 8:**
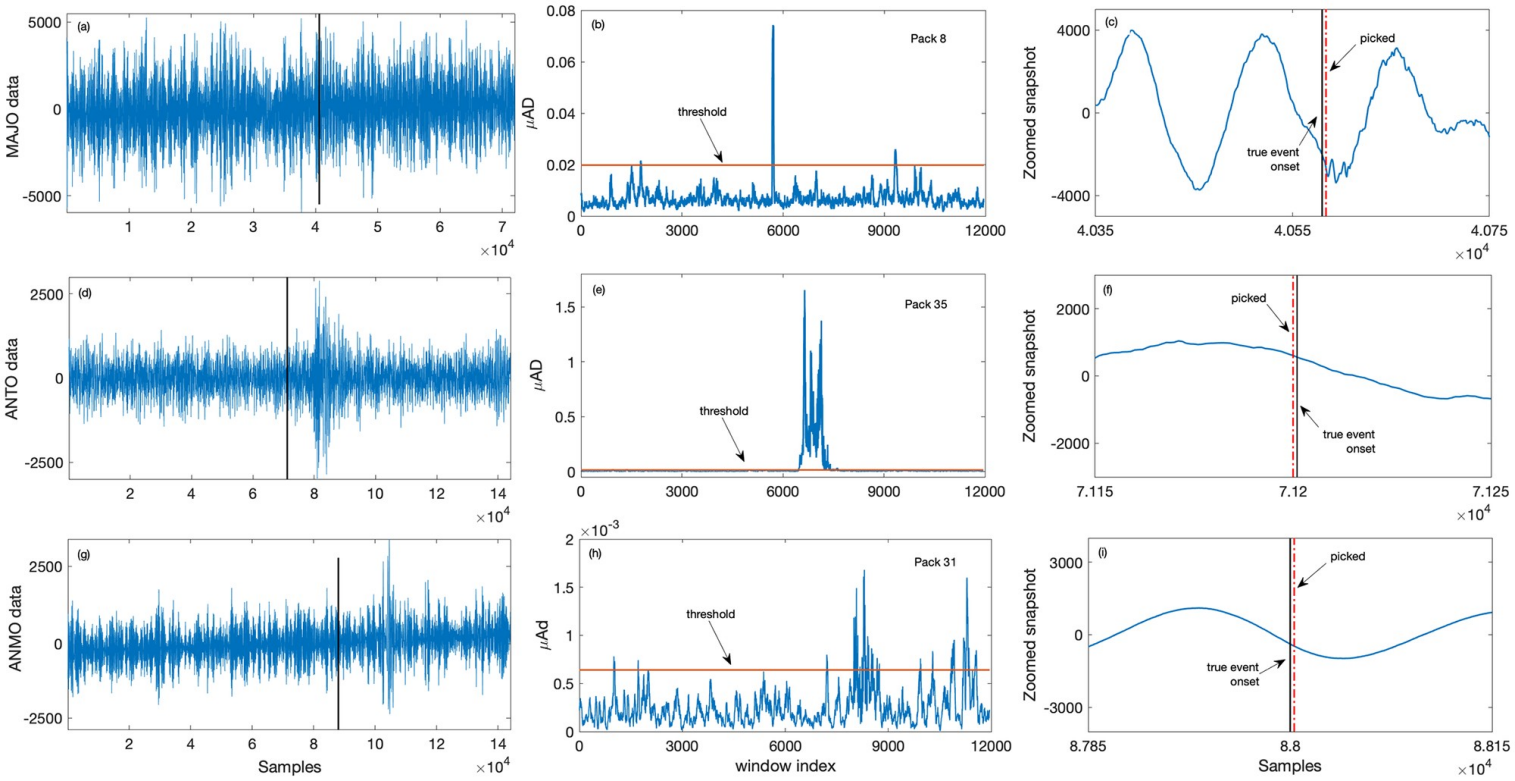
Event detection results for low SNR datasets. Plots (a), (d), (g) shows the vertical channel data from MAJO, ANTO, and ANMO station respectively, where the vertical black solid line indicates the onset of true event as reported on the IRIS website, plots (b), (e), (h) depicts corresponding *μ*AD in the selected packets. The solid horizontal line in the middle plots is the threshold value for the respective packets. Zoomed snapshots of respective data with picked events is shown in plots (c), (f) and (i) where the red vertical lines indicate the time at which the proposed method picks the event. Plots (c), (f), (i) shows the zoomed snapshot of data with picked events.

Vertical channel low magnitude (<2.5) and low SNR (<2) data acquired at different stations are shown in Fig 8(a) (SNR = −0.7), Fig 8(d) (SNR = 1.5), and Fig 8(g) (SNR = 0.001). Fig 8(b), 8(e) and 8(h) depict the mean absolute deviation corresponding to the packets 8, 35 and 31, respectively. As observed from Fig 8(c), 8(f) and 8(i) (zoomed snapshots of data), the algorithm picks the low magnitude and low SNR events with high accuracy (less than 0.2 sec) as compared to the onset time of event as reported on IRIS website.

For detection and picking purposes, an initial 10 min data is used for developing predictive models and computing threshold in the selected packets. Background noise at each station is modeled using ARIMA models of suitable orders. For instance, noise at ANMO is modeled using ARIMA(5, 1, 3), ANTO using ARIMA(4, 1, 6), MAJO using ARIMA(6, 1, 5), and TSUM using ARIMA(4, 1, 8) model. Both data and one-step-ahead predictions are decomposed into the time-frequency (t-f) domain using Daubechies 4 (db4) wavelet up to level 4 for 20 samples per second (sps) data and level 5 for 40 sps data. For most of the datasets, packets 8, 9, 16, 17, 18, 19, 21 are selected for 20 sps data. Additional to packets selected for 20 sps data, packets 27, 31, 32, 32, 33, 35 are also selected for 40 sps data. A sliding window (*w*_*n*_[*k*]) of length 240 samples with a sliding length (*S*) of 5 samples are used to detect the event. For accurate picking, a window of 1 sec with a sliding length of 1 sample is used in the detected segment.

**Fig 9:**
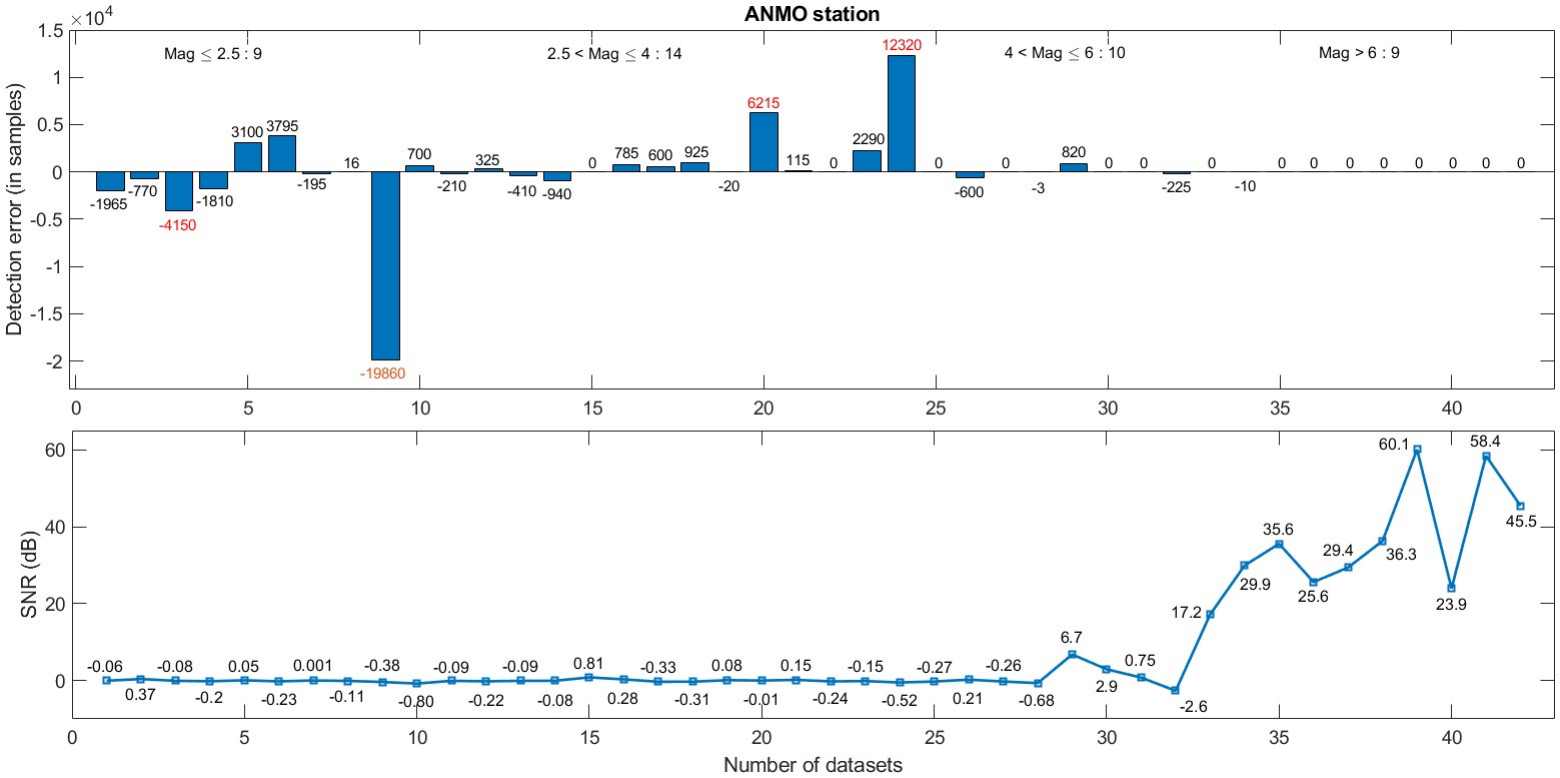
ANMO station: Event detection vs SNR. Top and bottom figures depict the detection error and SNR, respectively.

**Fig 10:**
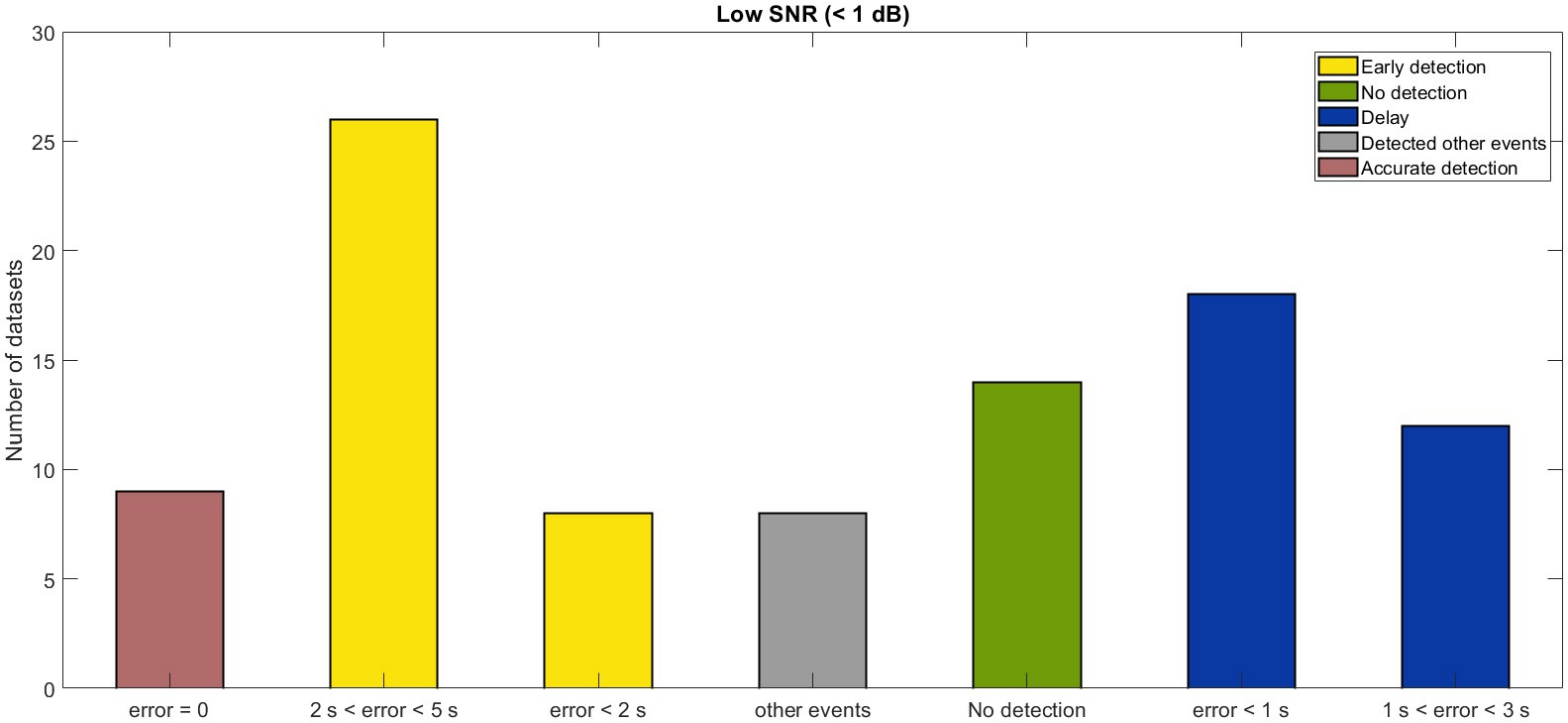
Detection rate for low SNR conditions. Detection rate of the proposed algorithm for SNR <5 dB. These datasets have events in the magnitude range from 0.13 to 4.

**Fig 11:**
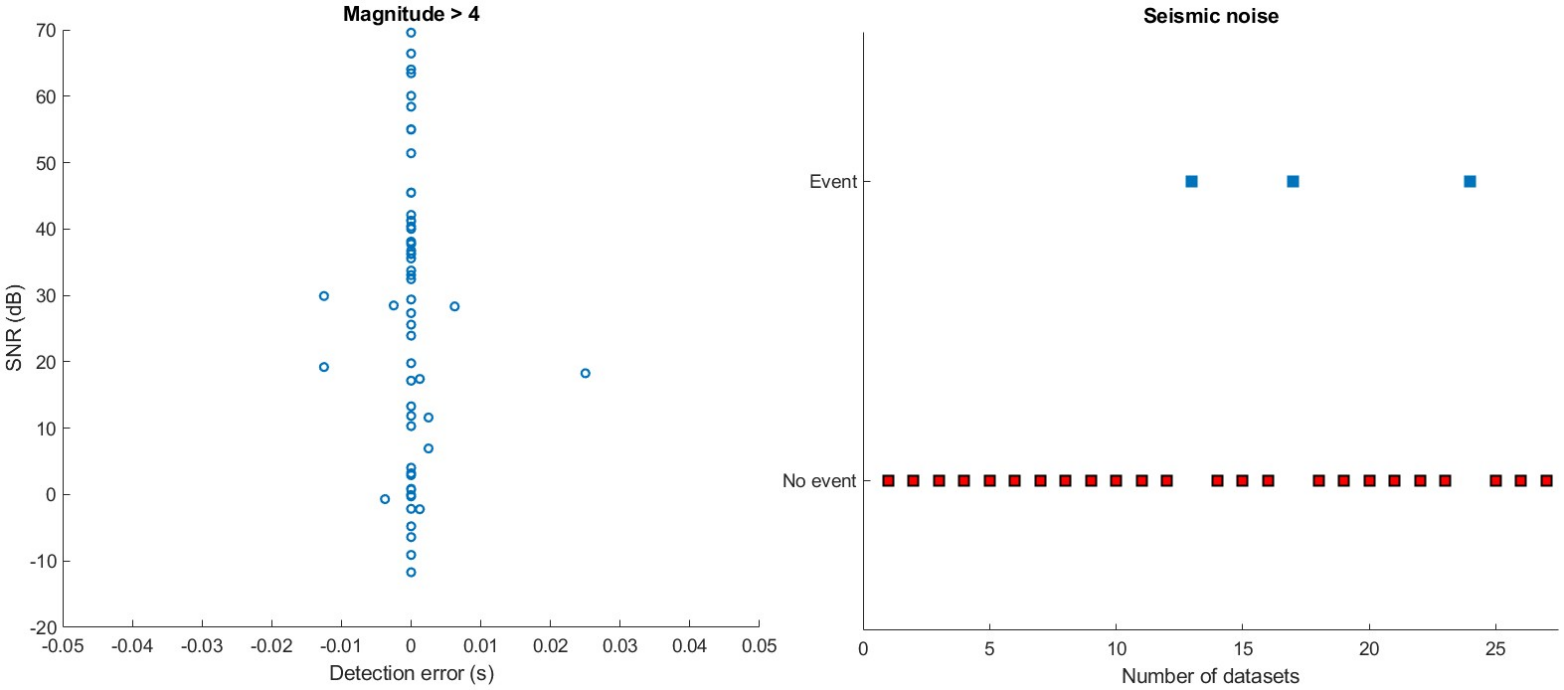
Detection error. The left plot depicts the detection rate of the proposed algorithm for high SNR >1 dB. The right plot shows the false alarm rate of the proposed method for background noise, where the red square box indicates no detection in the noise and blue boxes indicate false detection of events in background noise.

A comparative study of detected events with the true onset is carried out on all the 200 datasets to check the reliability of the proposed method. Fig 9 shows that the detection accuracy increases with the increasing SNR. The top figure depicts the detection error in different magnitude datasets downloaded from the ANMO station. The number on the top of each bar represents the detection error in samples, where a positive number indicates early detection and a negative number indicates delay. Numbers written in red color indicates the missed events. The corresponding SNRs are illustrated in the bottom plot of Fig 9. Furthermore, for ANMO station data, out of 42 events in Fig 9, the method fails to detect 4 events (bars with red color numbers on the top). For some datasets, the algorithm detects the event a bit earlier, despite the low SNR (<1 dB). A comparative study of different SNR data is also carried out to analyze the accuracy of the proposed algorithm for different SNR. Detection rate of the proposed method for low SNR (<1dB) is shown in Fig 10. Certain low magnitude datasets have multiple events in close proximity. For such datasets, the algorithm picks the stronger event among all the events (gray color bar in Fig 10). Fig 11 shows that the algorithm picks the high SNR events with high accuracy (<0.02 sec). For the no-event scenarios (background noise), the algorithm picks false events in 3 datasets out of 27, as shown in Fig 11.

**Fig 12:**
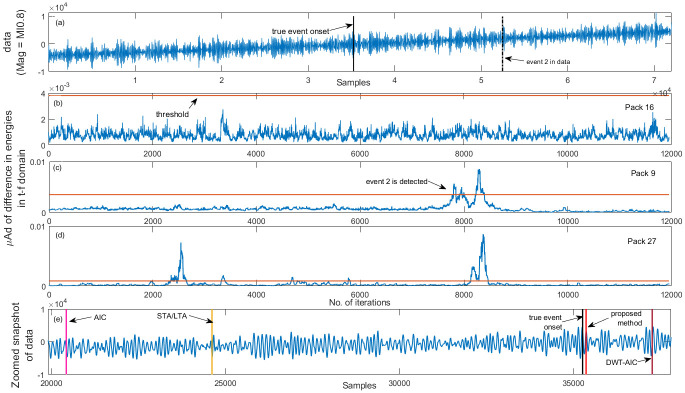
Low magnitude event detection results. Vertical channel TSUM station (a) data with two events—solid vertical black line indicates the true onset of the seismic event of interest and the dashed black color line indicates the second event in the data. Plots (b), (c) and (d) depict the *μ*AD corresponding to packets 9, 16 and 27 respectively and plot (e) is a zoomed snapshot of data—the red vertical line indicate the time at which the proposed algorithm picks the low magnitude event.

In order to illustrate the importance of packet (t-f band) selection, vertical channel data with more than one event from the TSUM station (mag Ml0.8) is selected. As shown in Fig 12(b), packet 16 fails to detect any of the events, packet 9 (Fig 12(c)) detects the second event accurately while missing the main event (of interest). However, as observed in Fig 12(d), packet 27 detects both the events with high accuracy. Therefore, if the user selects the wrong packets (t-f bands), the algorithm either fails to pick the event or miss it. As compared to the existing methods and the event time reported on the IRIS website, the algorithm successfully picks the extremely low SNR and low magnitude event with high accuracy (less than 2.5 sec).

**Fig 13:**
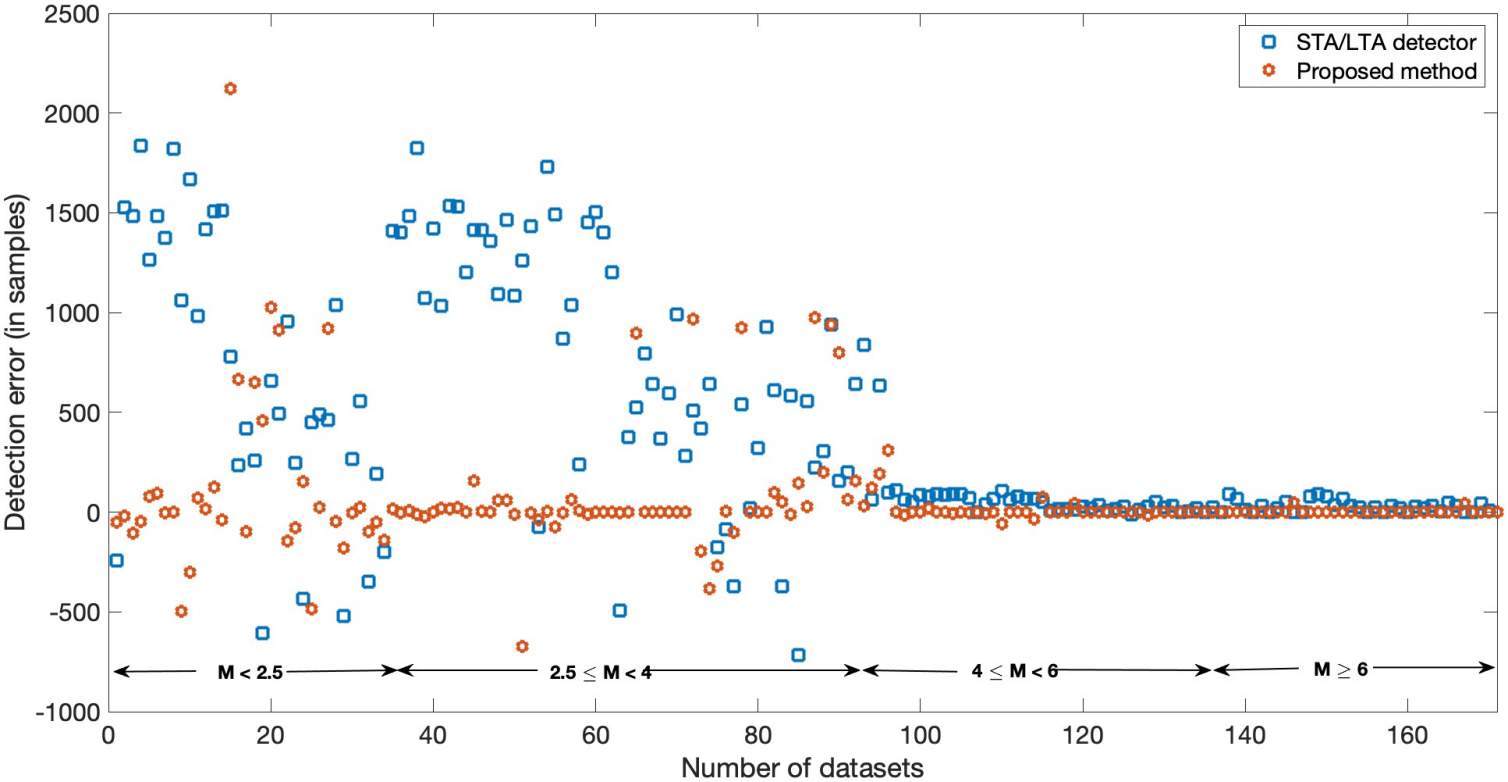
Comparison with existing detector. Detection error in samples of STA/LTA and the proposed method for events of different magnitude range.

**Fig 14:**
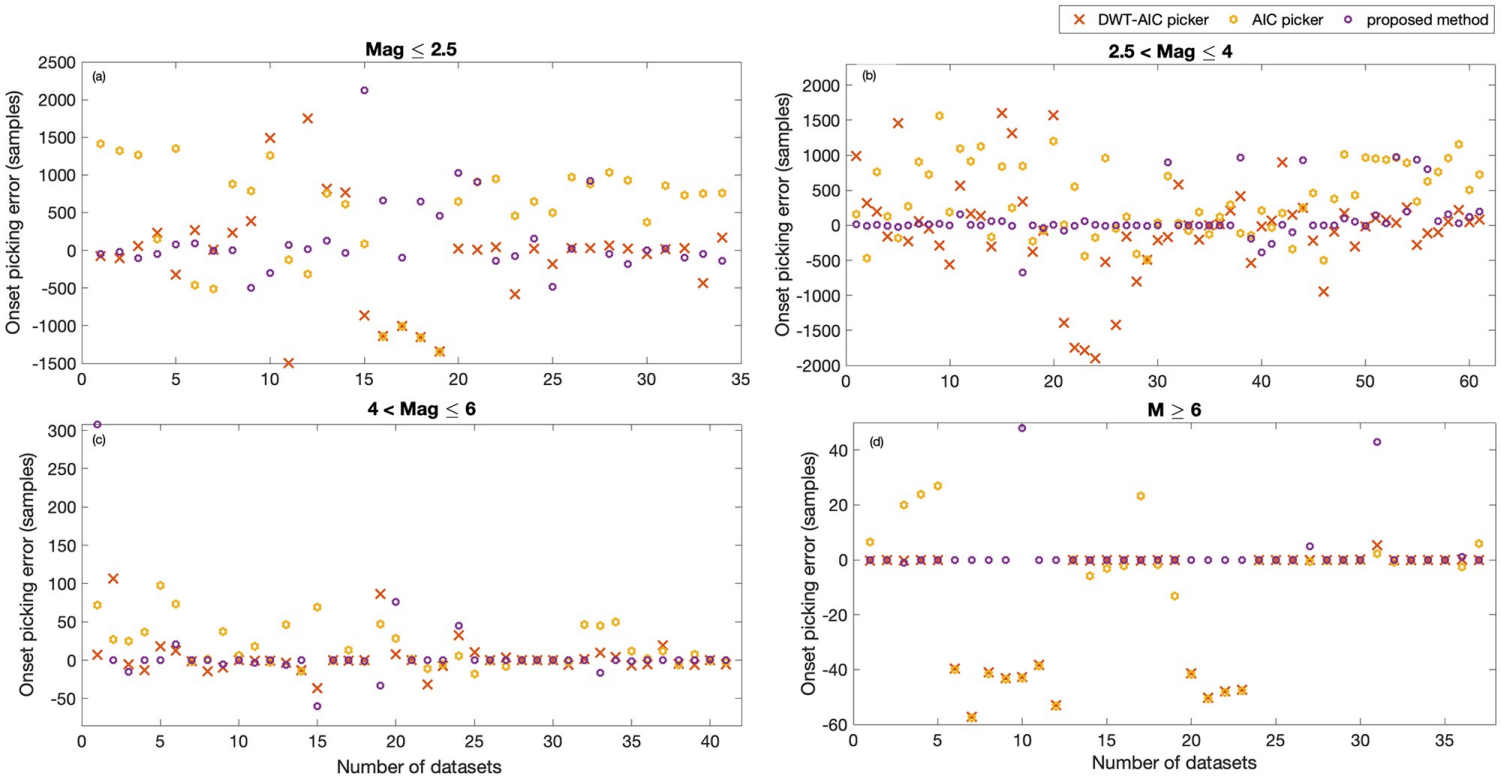
Comparison with existing pickers. Picking error in samples is reported for all the four categories, (a) Mag ≤2.5, (b) 2.5< Mag ≤4, (c) 4< Mag ≤6 and (d) Mag >6.

The proposed method’s performance is further evaluated by comparing it with the widely used STA/LTA detector, AIC, and DWT-AIC pickers. A set of user-defined parameters, such as window length, choice of wavelet, etc., govern the performance of each of these methods. These parameters are optimized for each station. For the STA/LTA detector, data is first filtered using a 4^th^ order Butterworth filter, with a passband of 0.2–2 Hz, before evaluating the characteristic function. The lengths of STA and LTA windows are fixed to 0.3 and 12 seconds, respectively. For the DWT-AIC picker, data is decomposed up to scale 7 using Daubechies 4 wavelet. It is observed from Fig 12(e) that both the STA/LTA detector and AIC pickers fail to detect or pick the low magnitude and low SNR event while the DWT-AIC picker picks the later phases (surface waves). Further, Figs 13 and 14(a) allow us to conclude that for events with magnitude <2.5, all the three existing methods fail to detect / pick the event onset. However, 20% of the later phases (surface waves) were detected by STA/LTA detector, 24% were picked by AIC picker, and 62% were picked by DWT-AIC picker. On the other hand, 20% of the P-wave arrivals in low magnitude events are picked with low accuracy, while 50% of the later phases were detected using the proposed method.

The detection rate of the proposed method is compared with the widely used STA/LTA detector while the picking rate is compared with the AIC and DWT-AIC pickers. Detection and picking errors of the proposed method and the existing detectors and pickers are depicted in Figs 13 and 14, respectively.

For the events of magnitude ranging from 2.5 to 4 (SNR <1 dB), STA/LTA fails to detect the P-wave onset in almost all the datasets (Fig 13). STA/LTA detects the P-wave arrival in only one out of the 61 datasets, however, around 20% of the later phases were detected. The proposed method, on the other hand, detects nearly 84% of the events with an accuracy of ≤2.5 sec. As shown in Fig 14, the widely used pickers also fail to pick most of the low magnitude and low SNR events. AIC and DWT-AIC pickers pick 3% and 8% of the P-wave arrival, respectively, with an accuracy of less than 5 sec. DWT-AIC picker picks 54% of the later phases while the AIC picker picks only 39%. The proposed method picks 56% of the P-wave arrival with high accuracy (<0.05 sec) while picking 33% with an accuracy of 2.5 sec. The proposed method fails to pick only 11% of the arrivals. For higher magnitude events (category 3: 4< Mag ≤6), all the methods detect or pick the P-wave arrival with a maximum error of 2.5 sec. However, around 14%, 24%, 54%, and 80% of the P-arrivals are detected/picked with an accuracy less than 0.05 sec by STA/LTA, AIC, DWT-AIC, and the proposed method, respectively. Figs 13 and 14(d) depicts that the higher magnitude events (Mag >6) are detected and picked by all the four methods with a maximum error of 1.25 sec. P-wave arrival is detected/picked accurately (with 0 error) in 10 datasets out of 34 by STA/LTA, 16 by AIC, and 22 by DWT-AIC picker. On the other hand, the proposed method accurately picks the P-wave arrival in 32 datasets.

**Fig 15:**
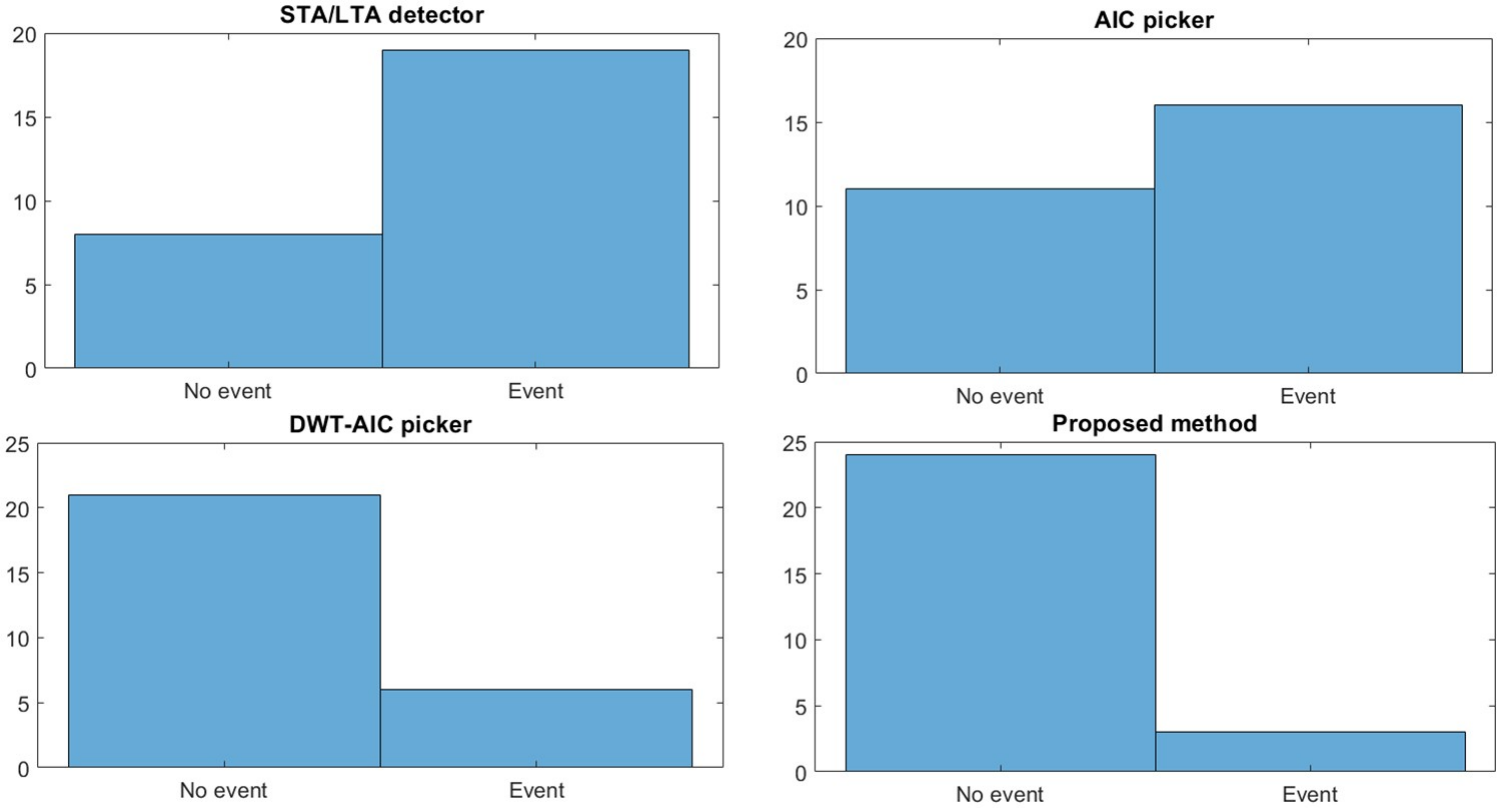
False alarm rate for different P-wave arrival detector / picker.

We have also compared the performance of these methods based on a false alarm rate, i.e., detecting an event when there is no event. Thus, all the three existing methods and the proposed method are implemented on seismic noise (event-free data). The 27 event-free datasets are carefully selected in the sense that we have considered impulsive noise (noise with impulsive spikes), noise during night time (less cultural activity), and daytime (cultural activities contribute significantly to noise) to study the robustness feature of the proposed framework. It is observed from Fig 15 that the proposed method outperforms the existing methods in that it results in lower false alarms than other methods.

Therefore the proposed method outperforms the existing methods by picking 63% of the P-wave arrivals with high accuracy (less than 0.05 sec) while detecting 27.6% of the arrival onset with an accuracy less than 2.5 sec. Table 5 summarizes the detection/picking accuracy and false alarm rate (FAR) of different methods. The proposed method outperforms the existing methods in both the metrics.

**Table 5:** Detection/Picking rate and false alarm rate in percentage. FAR stands for false alarm rate which is defined as the % of datasets with false detection of event.

Algorithm	% of datasets		
	≤ 0.05 sec	<5 sec	FAR
STA/LTA detector	8.23	30.59	70.37
AIC picker	14.11	40.59	59.25
DWT-AIC picker	23.5	51.76	22.22
Proposed framework	63	27.6	11.11

As discussed earlier, the performance of the proposed algorithm depends on certain data-driven and user-defined parameters. The following sub-section is devoted to the sensitivity analysis of these parameters.

### Crucial parameters

The performance of the proposed framework depends on the choice of parameters such as window length, sliding parameter (tuning parameters), threshold, etc. The influence of these parameters on the performance is discussed below.

**Fig 16:**
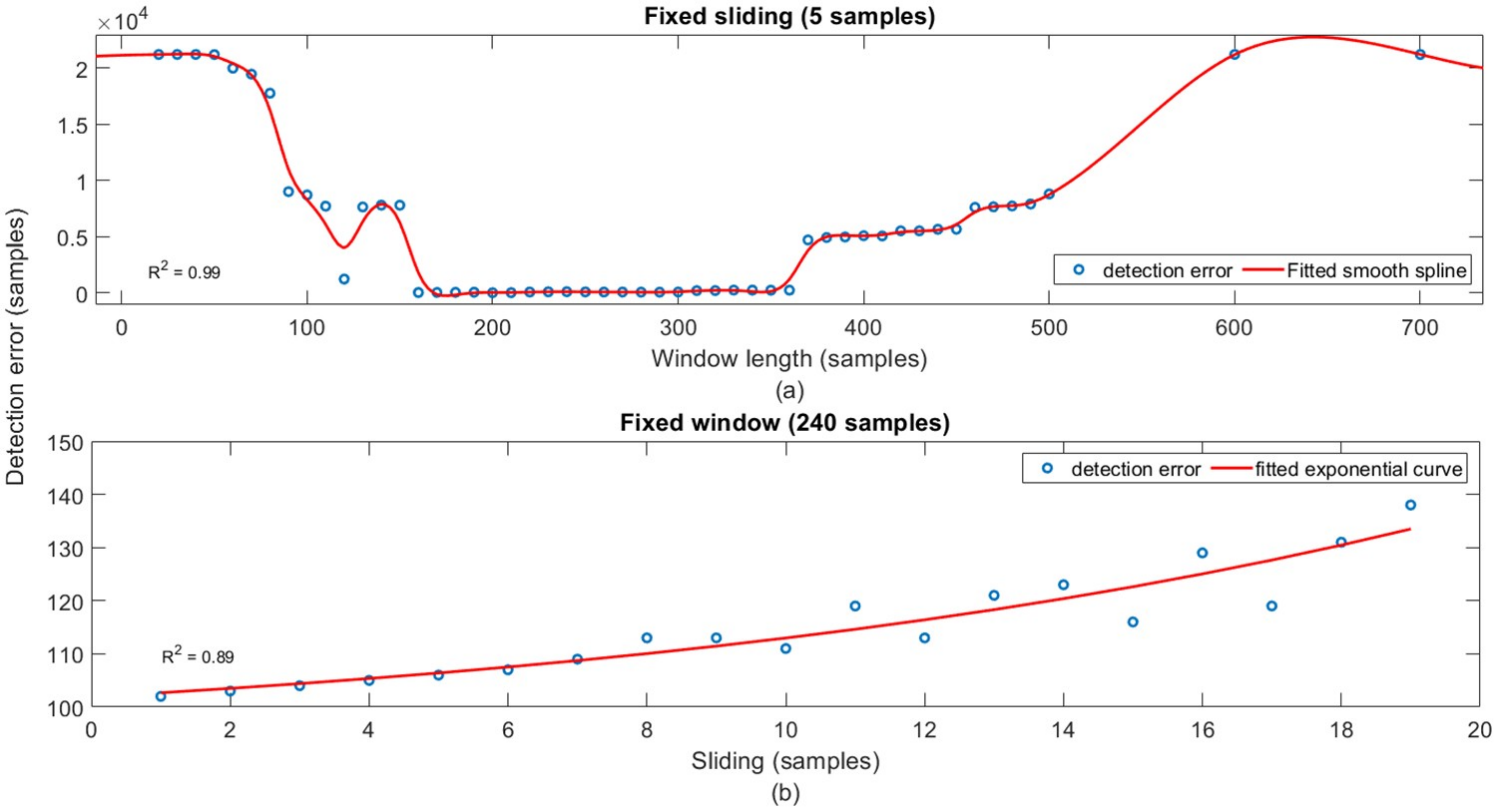
Sensitivity of crucial parameters. Effects of (a) varying window length for a fixed sliding length of 5 samples and (b) varying sliding length for fixed window length of 240 samples on the performance of the proposed method. Vertical axis indicates the detection error for varying tuning parameters. Blue color circles indicate the detection error, and the red color solid line is the fitted curve for both the parameters.

1. *Tuning Parameters*: Parameters such as window length and sliding parameter govern the performance of the detector and picker.
  - *Window length (N)*: The choice of a suitable window length for online detection and picking is one of the crucial steps. There exists a trade-off between the window length and detection rate. A shorter window with a small *N* results in increased type I error (false alarm rate; detecting events when there are no events) because of the increased sensitivity to the small changes in the data. On the other hand, selecting a wider window, larger *N*, reduces the type I error at the cost of increased type II error (detection rate; the probability of missing the event when there are events) because of the reduced sensitivity to the small changes. Therefore, it is necessary to select a window of suitable length that results in a low false alarm rate without compromising the detection rate.
  - *Sliding length (S)*: The choice of *S* depends on whether the purpose of the algorithm is to detect or to pick the event accurately. Larger *S* can be selected for detection purposes, while for precise picking of an event, a shorter *S* shall be used.
  The effects of these parameters on the performance of the proposed method are depicted in Fig 16. For illustration purposes, data from the ANMO station is considered. The detection error, indicated on the vertical axis of the plot, is defined as the difference between the true onset of the event and detected by the proposed method. As shown in Fig 16(a) initially, the detection error decreases with the increase in window length. However, after reaching the minima, the error increases with increasing window length. As observed from Fig 16(b), detection error increases with the increasing sliding length for a fixed window length of 240 samples.
2. *Threshold (**δ**)*: In this work, we use a data-driven static threshold computed offline from the historical event-free data (background noise). The threshold varies with t-f bands because each t-f band contains band-limited information. Therefore, using the same threshold for all the t-f bands result in erroneous detection.
3. *Level of decomposition (L)*: This parameter determines the total number of t-f bands in the t-f decomposition. *L* plays a vital role in determining the detection accuracy of the proposed framework. At a lower value of *L*, the bandwidth of t-f bands is higher, resulting in more contribution of noise and hence poor detection. However, if *L* is chosen to be high, the bandwidth of each t-f band reduces, resulting in the loss of the desired feature by splitting the information across several bands, thereby missing the event.

## Conclusion

This work presented a new prediction framework with a time-frequency localization feature to detect and pick the onset of P-wave in seismic measurements. The work is not only novel but is significant since it overcomes a key shortcoming of existing methods in handling low SNR measurements. Furthermore, it leverages the advantages of predictive analytics and the zoom-in feature of time-frequency transformation techniques. The main contributions of this work have been in (i) extending the applicability of predictive framework with t-f localization for efficient detection and picking of the seismic event, especially for low SNR cases, (ii) selecting suitable t-f bands on the fly to improve the detection accuracy, and (iii) optimally selecting the tuning parameters in order to reduce the false alarm rate. The devised picker provides an elegant detection and picking technique commensurate with the noise properties and is highly localized in the t-f domain. Therefore, it is robust to outliers and results in accurate detection for low SNR seismograms with minimal false alarms. The proposed method, by way of its construction, possesses certain desired features such as low false alarm rate, robustness to outliers, and, quite importantly, the ability to detect P-wave in low-quality seismograms. One of the most crucial steps that give the proposed method its ability to detect low SNR events is selecting the t-f bands, which is done through a ranking procedure. Finally, the use of variability in the difference of energies between the data and predictions in the t-f domain as a statistic to conduct the test of detection makes it sensitive to the arrival of P-wave. An added benefit of the proposed method that has significant potential in mathematical modelling of P-waves is that it facilitates the reconstruction of P-wave signatures from seismic measurements, which has not been addressed before in the reported literature.

We have demonstrated the proposed framework using ARIMA models as predictive vehicles and the maximal overlap DWT for time-frequency projections of measurements and predictions. The ARIMA models were built using a systematic procedure developed by the authors and reported elsewhere. Implementation on nearly 200 datasets with diverse characteristics showed that the proposed framework is not only robust to outliers (low false alarm in the noisy data) but is also capable of detecting the extremely low SNR (<0.13) and low magnitude (<0.9) events. The proposed framework outperforms widely used STA / LTA, AIC, and DWT-AIC methods, especially for low SNR events.

The success of the proposed framework and its ability to detect under extremely low SNR conditions should not be construed as a coincidence since the success of the proposed method rests on the band-limited SNR and not on the conventional SNR, a widely used yardstick. The band-limited SNR is high even as the standard SNR is very low for the events under analysis. This is due to the significant differences in the time-frequency distributions of the event and seismic noise characteristics (multi-scale nature of the seismic measurement). By their construction, existing methods do not exploit the high-value of band-limited SNRs or rather the multi-scale nature of data and hence fail to perform under low SNR scenarios. Any method that zooms into the t-f characteristics of the data potentially stands to benefit from the band-limited SNR. Adding a layer of predictions and comparing the differences in characteristics between the two layers (data and predictions) significantly improves the discriminating ability of the detection technique. This essentially is the core idea of the proposed framework and rationalizes the benefits it brings in to the problem of interest.

## Supporting information

S1 File (PDF)
