# Supplementary material for: A prediction framework with time-frequency localization feature for detecting the onset of seismic events
Source: PLoS One. 2021 Apr 22;16(4):e0250008. doi: 10.1371/journal.pone.0250008 (PMC8062077; doi:10.1371/journal.pone.0250008)
Supplement: S1 File — (PDF) [file pone.0250008.s001.pdf]

S1 Appendix. Distribution of $\mathbf{E}_d$ In the nth window, $\mathbf{E}_d(l, \omega_{c,i})$ is the difference between the squared absolute magnitudes of projections of measurements and predictions in the ith t-f band at level l. In a separate study by the authors, it has been statistically determined that the seismic noise follows Gaussian distribution. Therefore the random variables $\mathbf{E}_{\mathbf{w}_n}$ and $\mathbf{E}_{\hat{\mathbf{w}}_n}$ follow a $\chi^2(\nu)$-distribution with $\nu < N$ degrees of freedom (due to correlation in seismic noise). $\mathbf{E}_d$ is the difference between two χ^2 distributed random variables. The histograms of $\mathbf{E}_d$ in packets 8 and 18 are shown in S1 Fig A. Although a visual analysis of the histogram suggests a Gamma distribution, in order to arrive at the best distribution fit, we use the Cullen and Frey graph for $\mathbf{E}_d(3, \omega_{c,2})$ and $\mathbf{E}_d(4, \omega_{c,4})$ shown in S1 Fig B. It indicates that $\mathbf{E}_d$ follows a negative binomial distribution, which is the ratio of two Gamma distributions. This is also in agreement with the findings in the literature on the distribution of the difference between two χ^2 distributed random variables. There exists no work though that directly computes the correction factor for a negative binomial distribution. Therefore, the correction factor for μAD has to be calculated numerically using Monte-Carlo simulations. For this purpose, μAD for negative binomial distributed random variable is estimated using 500 realizations and averaged to compare with the standard deviation σ. S1 Fig C shows that applying a factor of 1.25 to μAD results in an unbiased estimate of σ for the negative binomial distributed random variable. Note that the simulations generate the negative binomial distributed variable as the difference of two χ^2-distributed random variables with $\nu = N = 240$ degrees of freedom. The correction factor, although determined for this setting, is found to be invariant over a wide range of degrees of freedom. Therefore, we set the correction factor for μAD as $K = 1.25$.

S1 Fig A. Histogram of $\mathbf{E}_d$ in packet 8 (level 3) and packet 18 (level 4).

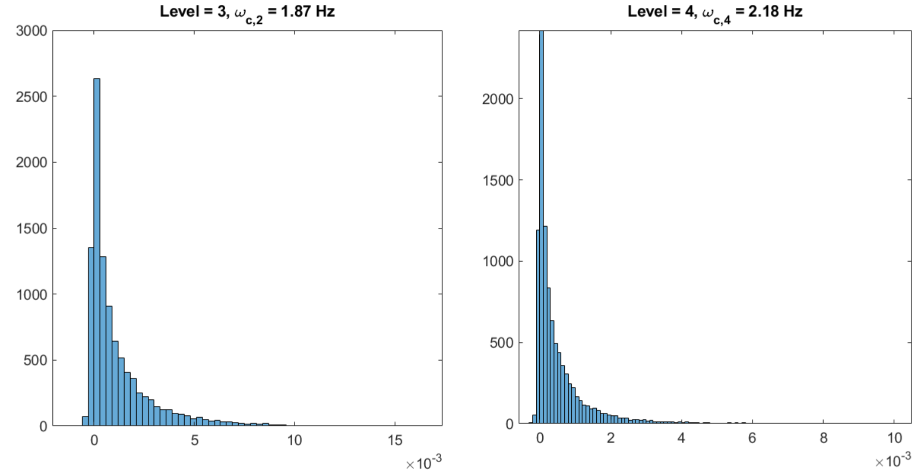

S1 Fig B. CF graph of $\mathbf{E}_d$ in packet 8 (level 3) and packet 18 (level 4).

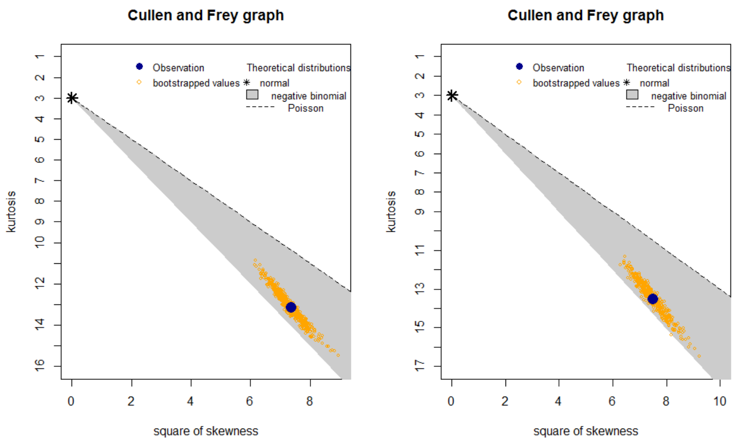

S1 Fig C. **Estimates of variability** for negative binomial distributed random variable.

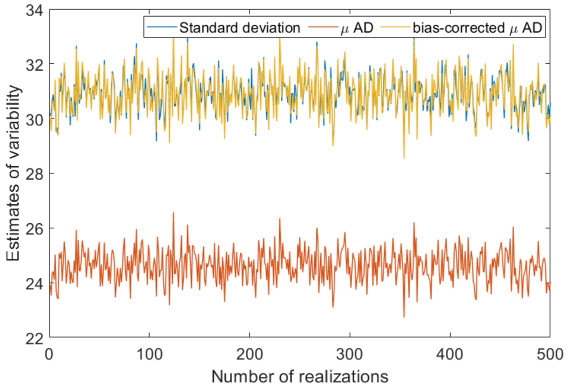

S2 Data. **Data Availability** All the datasets used in this study are publicly available on the Incorporated Research Institution of Seismology (IRIS). Its data management center archives and distributes data in order to conduct seismological research. All the datasets can be downloaded from IRIS: Wilber 3: Select event by specifying the details of event and station.
